# *OsCER1* Plays a Pivotal Role in Very-Long-Chain Alkane Biosynthesis and Affects Plastid Development and Programmed Cell Death of Tapetum in Rice (*Oryza sativa* L.)

**DOI:** 10.3389/fpls.2018.01217

**Published:** 2018-09-06

**Authors:** Erdong Ni, Lingyan Zhou, Jing Li, Dagang Jiang, Zhonghua Wang, Shaoyan Zheng, Hua Qi, Ying Zhou, Cimei Wang, Shi Xiao, Zhenlan Liu, Hai Zhou, Chuxiong Zhuang

**Affiliations:** ^1^State Key Laboratory for Conservation and Utilization of Subtropical Agro-Bioresources – Key Laboratory of Plant Functional Genomics and Biotechnology of Guangdong Provincial Higher Education Institutions, College of Life Sciences, South China Agricultural University, Guangzhou, China; ^2^Laboratory Center of Basic Biology and Biotechnology, Education Department of Guangdong Province, Zhongkai University of Agriculture and Engineering, Guangzhou, China; ^3^Key Laboratory of Crop Stress Biology for Arid Areas, College of Agronomy, Northwest A&F University, Yangling, China; ^4^State Key Laboratory of Biocontrol and Guangdong Provincial Key Laboratory of Plant Resources, School of Life Sciences, Sun Yat-sen University, Guangzhou, China

**Keywords:** *Oryza sativa* L., male sterility, plastid development, tapetum degeneration, anther cuticle wax

## Abstract

Cuticle waxes, which are primarily comprised of very-long-chain (VLC) alkanes, play an important role in plant reproductive development. *ECERIFERUM1* (*CER1*) is recognized as the core element for VLC alkane biosynthesis in Arabidopsis (*Arabidopsis thaliana*). However, genes involved in the VLC alkane biosynthesis in rice remain unclear, and the alkane-form pathway in rice has still to be further explored. Here, we show that *OsCER1*, a homology of *CER1*, functions in VLC alkanes biosynthesis, which also could regulate anther development and plastids differentiation in rice. *OsCER1* was highly expressed in the tapetum (stage 10) and bicellular pollen cells (stage 11). The decreased content of VLC alkanes (C25 and C27) in the *OsCER1* knocked down plants as well as the increased content of C27 alkanes in the *OsCER1* overexpression plants indicates that *OsCER1* participates in VLC alkane biosynthesis. Downregulation of *OsCER1* in rice led to sterility, and fewer amyloplasts within the mature pollen grains. In addition, the downregulation of *OsCER1* in rice caused delayed tapetal programmed cell death and abnormal development of plastids in the tapetal cells. Furthermore, significantly altered levels of expression of genes involved in the pollen development were exhibited in the *OsCER1* knocked down plants. These results indicate that *OsCER1* is critical for VLC alkanes biosynthesis, plastids differentiation, and pollen development. This work provides insights into the VLC alkanes biosynthesis in anther development in rice.

## Introduction

In flowering plants, successful male reproductive development within the anther includes critical developmental events such as meristem specification, sporogenous cell differentiation, meiosis, and mitosis (Scott et al., 2004; Blackmore et al., 2007; Wilson and Zhang, 2009). Rice (*Oryza sativa* L.) is one of the most important food crops in the world. Abnormal reproductive development has significantly influenced rice production. Rice anthers have four lobes in which the primordial cells differentiate into reproductive pollen mother cells. Four somatic cell layers surround these cells: the tapetum, the middle layer, the endothecium, and the epidermis, which are arranged from the innermost to the outermost layer (Goldberger et al., 1995; Zhang and Yang, 2014).

Covering the epidermal cells, the rice anther has a reticulate cuticle, which plays a protective role in pollen development (Li and Zhang, 2010). The anther cuticle is a thin, continuous hydrophobic layer that coats the outermost surface of anthers and is mainly composed of epicuticular waxes and a cutin matrix (Kunst and Samuels, 2003; Jung et al., 2006; Nawrath, 2006; Pollard et al., 2008). Cuticular waxes are mostly composed of very-long-chain fatty acids (VLCFAs) derivatives, including primary and secondary alcohols, aldehydes, alkanes, ketones, and esters (Kunst and Samuels, 2003; Buschhaus and Jetter, 2011). Abnormal cuticular waxes of anthers frequently accompany aborted pollen development (Jung et al., 2006; Jessen et al., 2011; Leide et al., 2011; Smirnova et al., 2013; Haslam et al., 2015).

The biosynthesis of VLCFAs occurs in two stages. First, *de novo* fatty acids (FAs), such as C16 and C18 FAs, are synthesized in the plastid stroma and are catalyzed by the FA synthase complex using acetyl-CoA as the starting substrate. The majority of long-chain fatty acids (LCFAs) serve as central intermediates in catalytic steps, such as elongation and acyl editing (Haslam and Kunst, 2013), and some LCFAs remain in plastids integrated into polar lipids, such as glycolipids and phospholipids (Li-Beisson et al., 2013).

The second stage of VLCFA biosynthesis is the elongation of C16 and C18 FAs into VLCFAs consisting of C20 to C34 chains. The synthesis of VLCFAs is catalyzed by fatty acyl-CoA elongase in the endoplasmic reticulum (ER) (Hooker et al., 2002; Efremova et al., 2004; Bach et al., 2008; Joubes et al., 2008; Beaudoin et al., 2009). Subsequently, extended VLCFAs are modified into diverse VLCFAs derivatives, the main components of wax, via two pathways, namely, the alcohol-forming (acyl reduction) pathway and the alkane-forming (decarbonylation) pathway. The alcohol-forming pathway is involved in the production of very-long-chain (VLC) primary alcohols catalyzed by a fatty acyl-CoA reductase. The alkane-forming pathway yields VLC alkanes, aldehydes, secondary alcohols, and ketones (Jenks et al., 1995; Rashotte et al., 2001). Among these metabolites, VLC alkanes predominate in the wax structure, and account for 50–70% of the total wax content in numerous different plants species (Kunst and Samuels, 2003; Kosma et al., 2009; Kunst and Samuels, 2009). The VLC alkanes play critical roles in various plant physiological processes, notably in plant fertility and resistance to drought stress (Aarts et al., 1995; Kosma et al., 2009; Bourdenx et al., 2011).

Recently, several genes function in the metabolism of VLCFAs and wax were discovered (Kunst and Samuels, 2003; Buschhaus and Jetter, 2011), and several *eceriferum* (*cer*) mutations in Arabidopsis affect wax composition, suggesting that CER proteins play important roles in wax biosynthesis (Jenks et al., 1995; Lai et al., 2007). For instance, CER1 and CER3, which are localized in the ER, may form a complex that catalyzes the synthesis of VLC alkanes from VLC acyl-CoAs via a two-step reaction (Bernard et al., 2012). The *GLOSSY1 (GL1)* gene in maize (*Zea mays*), a homolog of *CER3*, affects the biosynthesis of epicuticular waxes on the surfaces of seedling leaves (Sturaro et al., 2005). *CsCER1* and *CsWAX2* in *Cucumis* are involved in the biosynthesis of VLC alkanes and influence pollen fertility (Wang et al., 2015a,b). It is important to understand the VLC biosynthesis pathway in rice at the molecular level because rice is one of the most important food crops worldwide. Recent studies have reported that *Wax Crystal-Sparse Leaf 2* (*WSL2*), *OsGL1-2, OsGL1-3, Wax-deficient Anther 1* (*WDA1*), and *OsGL1-6* are homologs of *CER* involved in the wax biosynthesis in rice (Jung et al., 2006; Qin et al., 2011; Mao et al., 2012; Zhou et al., 2013, 2015). However, the alkane-form pathway in rice still needs to be explored, and whether other unreported *CER* homologs in rice are involved in VLC alkanes biosynthesis remains unclear.

Plastids where *de novo* fatty acids synthesis occurred play crucial roles in successful anther development. During anther development, proplastids in tapetal cells undergo division during the early stages of microsporogenesis and subsequently develop into elaioplasts that are associated with biosynthesis of the lipids that tapetum cells secrete into the anther locule to contribute to pollen wall formation (Clément and Pacini, 2001). For instance, No Exine Formation 1 (NEF1) is a plastid integral membrane protein that plays an important role in tapetum degeneration, plastid development, and lipid accumulation, and effects fertility in Arabidopsis (Ariizumi et al., 2004). However, whether there are lipid signals from plastids that regulate anther development and how these pathways accomplish this regulatory role remain unclear.

In this article, we isolated a homologous protein of CER1, OsCER1, and characterized its function in rice. *OsCER1* is preferentially expressed in tapetum cells at stage 10 and in bicellular pollens at stage 11 of anther development. We obtained several independent *OsCER1* downregulated lines, and found that these plants displayed low fertility, including abnormal anther development with smaller and fewer amyloplasts within the mature pollens. We also observed abnormal plastids differentiation in the tapetum. Furthermore, we provide evidence for the pivotal role of *OsCER1* in the biosynthesis of VLC alkanes and in the tapetum degeneration during anther development in rice.

## Materials and Methods

### Plant Materials and Growth Conditions

The rice variety Zhonghua 11 (*Oryza sativa* L. ssp. *japonica* cv. Zhonghua 11) was used for all experiments in this study. The wild-type (WT) and transgenic plants were grown in a paddy field at South China Agricultural University. The developmental stages of the spikelets were determined according to spikelet length and anther morphology as described by Zhang et al. (2011).

### Phylogenetic Analysis

Sequence alignment of OsCER1 and its related proteins from rice and other species was performed using Clustal Omega from EMBL-EBI^1^ to construct a phylogenetic tree, using default parameters. The phylogenetic tree was constructed using the neighbor-joining method as implemented in the MEGA program version 3.0 with 1,000 bootstrap replications^2^. CER1 and OsCER1 were aligned with ClustalW^3^, followed by manual alignment in conserved active His-rich motifs.

### Generating *OsCER1* Antisense-RNA and Overexpression Transgenic Plants

The *OsCER1* antisense transgene driven by the *OsCER1* promoter was constructed as described by Li J. et al. (2011). A 560-bp fragment of the *OsCER1* cDNA was amplified using reverse transcription-polymerase chain reaction (RT-PCR) from Zhonghua 11 with gene-specific *OsCER1*-A F/R primers (**Supplementary Table 4**). The PCR product was inserted in reverse orientation into the multiple cloning site of pCAMBIA1380. A 2,036-bp genomic fragment located upstream of the annotated ATG start codon of *OsCER1* was isolated from Zhonghua 11 genomic DNA using *OsCER1*-P F and *OsCER1*-P R primers (**Supplementary Table 4**) and then inserted upstream of the antisense fragment. A 1,860-bp ORF of *OsCER1* cDNA was amplified using RT-PCR from Zhonghua 11 with gene-specific *OsCER1*-OE F/R primers (**Supplementary Table 4**) and inserted into the multiple cloning site of pYLpox driven by *Ubi* promoter. Then constructs were transformed into *Agrobacterium tumefaciens* strain EHA105. Transgenic plants were obtained as described by Li J. et al. (2011). Two homozygous *OsCER1* antisense transgenic (*OsCER1A*) lines, *OsCER1A* 3-6 and *OsCER1A* 9-1, and two homozygous *OsCER1* overexpression transgenic lines, *OsCER1* overexpression 3 (OV-3) and *OsCER1* overexpression 6 (OV-6) were obtained and used for relevant analysis.

### Southern Bolt and Quantitative Reverse Transcription-PCR (qRT-PCR) Analysis

Genomic DNA was extracted with the plant genomic DNA miniprep Kit (Sigma, United States) from the 3–5 g mature leaves of WT and the nine *OsCER1* antisense-RNA transgenic lines, respectively. To generate a *Hpt* gene-specific probe for the Southern bolt, a 651-bp *Hpt* fragment was amplified using the *Hpt* F and *Hpt* R primers (**Supplementary Table 4**) and labeled with a PCR DIG labeling mix (Roche, Diagnostics). The Southern bolt procedures were performed as previously described by Ariizumi et al. (2004). Total RNA was isolated from rice tissues using TRIzol reagent (Invitrogen, United States), including shoots, roots, and spikelets with anthers at different stages. First-strand cDNA synthesis was performed using a Takara PrimeScript first-strand cDNA synthesis kit (Takara Bio Inc.), and the cDNA was then used for qRT-PCR, which was performed in triplicate using SsoFast EvaGreen Supermix (Bio-Rad, United States). All primers for qRT-PCR are listed in **Supplementary Table 4**. *OsActin1* was used as reference for normalization of mRNA expression.

### RNA *in situ* Hybridization

Rice anthers from stages 6 to 11 were collected. To generate gene-specific and control probes, a 613-bp *OsCER1* fragment was amplified using the *OsCER1* HIS F and *OsCER1* HIS R primers (**Supplementary Table 4**) and inserted into a pEASY-Blunt vector (Transgen, Beijing, China). The probes were transcribed *in vitro* from the T7 promoter using RNA polymerase as provided in the DIG RNA labeling kit (Roche, Indianapolis, IN, United States). The following RNA *in situ* hybridization procedures were performed as previously described by Kouchi and Hata (1993).

### Terminal Deoxynucleotidyl Transferase-Mediated dUTP Nick-End Labeling (TUNEL) Assay

For the TUNEL assay, 6-μm sections of anthers were prepared and the following procedures were performed using the TUNEL apoptosis detection kit (DeadEnd Fluorometric TUNEL System, Promega). Slides were stained with propidium iodide (PI). Green GFP signals (excitation wavelength: 488 nm) and the red PI signals (excitation wavelength: 543 nm) were assessed using a confocal laser-scanning microscope (LSM510 Mera; Carl Zeiss).

### Microscopy Analysis

Anthers from mature flowers were dissected, and pollen grains were stained with iodine/potassium iodide (I_2_/KI) staining (0.2% iodine and 2% potassium iodide) and observed under a brightfield microscope (Zeiss, Axio Observer D1, Germany). To investigate pollen germination *in vitro*, mature pollen grains were germinated on a glass slide at 33°C for 30 min in a pollen germination medium composed of 2.0% (m/v) agar, 30% (m/v) sucrose, 50 mM KNO_3_, 400 mM H_3_BO_3,_ and 400 mM MgSO_4_⋅7H_2_O. The pollen grains were then observed under a brightfield microscope (Zeiss, Axio Observer D1).

Anthers of the WT and *OsCER1A* plants at different stages were fixed in 3.7% formaldehyde/acetic acid (v/v) and resin-embedded semi-thin sections (epoxy resin PolyBed 812, Polysciences) were made and observed as described by Zhu et al. (2011). For transmission electron microscopy (TEM), anthers were fixed in sodium phosphate buffer (pH 7.2) containing 4% (w/v) paraformaldehyde and 2.5% (v/v) glutaraldehyde and sectioned as described by Schulz et al. (1993). The sections were observed using an electron microscope (TECNAI G2 12, FEI) at 100–120 kV. For scanning electron microscopy (SEM), anthers were fixed in sodium phosphate buffer (pH 7.2) containing 4% (w/v) paraformaldehyde and 2.5% (v/v) glutaraldehyde. The following SEM procedures were performed as previously described by Zhou et al. (2015).

### Subcellular Localization

The *CaMV35S* promoter, *eGFP* fragments, and *NOS* terminator were successively cloned into pUC18 to construct the *eGFP* transient expression (*GFP*) vectors. The signal peptide fragment of *OsCER1* was amplified using the primer pair *OsCER1-eGFP F, OsCER1-eGFP R* (**Supplementary Table 4**) and then subcloned into the *GFP* vector between the *CaMV35*S promoter and the *eGFP* gene, in-frame with the N-terminus of *eGFP* being driven by the *CaMV35S* promoter. Protoplasts from the leaf sheaths of 14-day-old rice seedlings were prepared, and transient expression was conducted following the protocol described by Zhou et al. (2015).

### WAX Profiling

For the anthers wax composition studies, waxes were extracted from 10 to 20 mg of freeze-dried anthers and analyzed as described by Zhou et al. (2015). For the leaves wax composition analysis, 20–50 mg of freeze-dried flag leaves were harvested in a Teflon-lined screw cap glass tube, extracted, and analyzed following the method of Zhou et al. (2015).

### Accession Numbers

The sequences used in this study were downloaded from GenBank with the following accession numbers: *CER1* (D64155.1), *CER3* (X95962), *BnCER1* (KF724897.1), *CsCER1* (KJ461885), *CYP704B2* (Os03g07250), *Defective Pollen Wall* (*DPW*) (EU971135), *GL1* (U37428.1), *Rice ATP binding cassette subfamily G 15/ Post-meiotic deficient anther 1* (*OsABCG15/PDA1*) (Os06g40550), *OsC*6 (Os11g37280), *OsNEF1* (Os11g32470), *OsGL1-3* (AK070469), *WSL2* (AK060786), *OsGL1-2* (AK066569), *OsCER1* (AK066386), *WDA1* (AK100751), *OsGL1-6* (AK068166), Os*RAFTIN1* (Os08g38810), *Tapetum Degeneration Retardation* (*TDR*) (Os02g02820), and *TDR Interacting Protein 2* (*TIP2*) (Os01g18870).

## Results

### *OsCER1* Is Homologous to *CER1*

**FIGURE 1:**
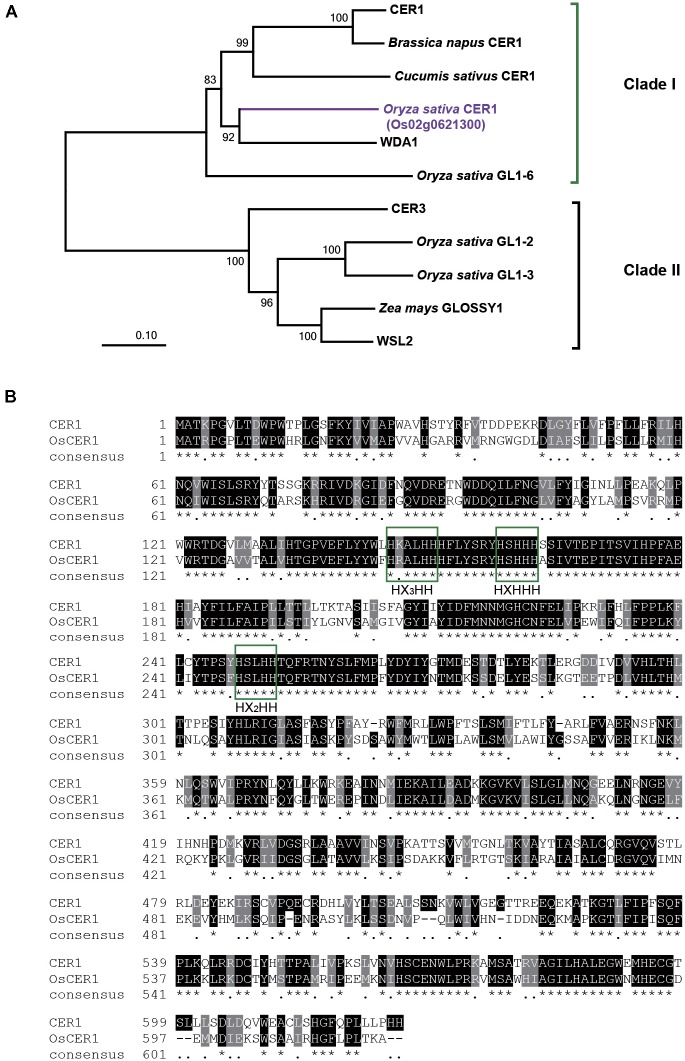
Phylogenic analysis and sequence alignment of OsCER1. **(A)** Phylogenetic analysis of the OsCER1 protein and 10 OsCER1 related proteins. Bootstrap values are shown on the branches. The scale bar represents the number of amino acid substitutions per site. The value of 0.01 on the scale bar indicates 1 amino acid substitution per 100 residues. The numbers near the branches refer to the bootstrap value of the neighbor-joining phylogenetic tree. The length of the branches is proportional to the amino acid variation rates. **(B)** Sequence alignment between CER1 and OsCER1. Identical and similar residues are shown on black and gray backgrounds, respectively. Green boxes indicate the three conserved active His-rich motifs; asterisks (^∗^) indicate the identical residues in all sequences, black dots (.) indicate the semi-conserved substitutions, and dashes (–) indicate gaps in the alignment.

Phylogenetic reconstruction indicated that CER1 and other 10 homologous proteins of CER1 could be classified into two clades; CER1 and CER3 belonging to clades I and II, respectively (**Figure 1A** and **Supplementary Data Set 1**). The gene Os02g0621300 located on rice chromosome 2 is a homology of *CER1*, thus we named this gene *OsCER1*. The full-length 2,481-bp cDNA clone of *OsCER1* contains an open reading frame of 1,860 bp in length, which encodes a 619-amino acid protein (XP_015627618.1) with a molecular mass of 71 kD. Putative conserved domains include a fatty acid hydroxylase superfamily domain (amino acids 139–253) and a WAX2 C-terminal domain (amino acids 453–615) (Islam et al., 2009). OsCER1 was grouped into clade I, and shared 57% sequence identity with CER1. Alignment of CER1 and OsCER1 showed that their predicted amino acid sequences contained the same three conserved histidine-rich (His-rich) motifs (**Figure 1B**) that are likely critical for alkane synthesis function (Bernard et al., 2012).

### *OsCER1* Is Mainly Expressed in Tapetum and Microspores

**FIGURE 2:**
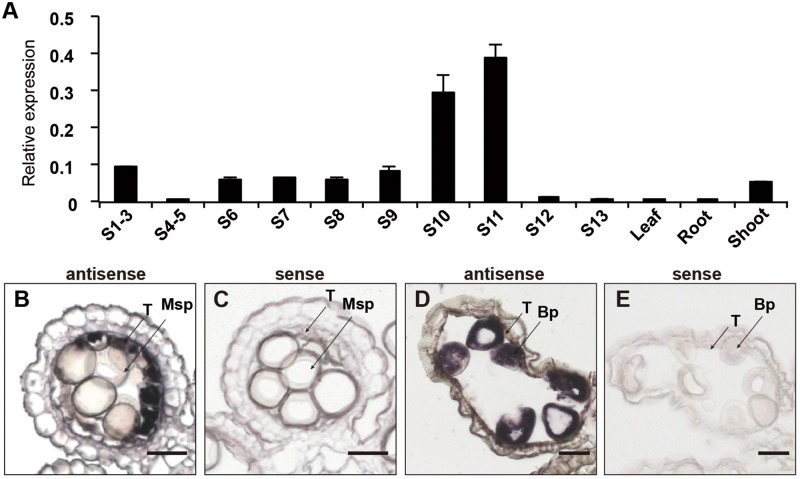
The expression patterns of *OsCER1*. **(A)** Spatial and temporal expression analysis of *OsCER1* in spikelets at various anther developmental stages by qRT-PCR. S1-3, before stage 3; S4-5, from stages 4 to 5; S6, stage 6; S7, stage 7; S8, stage 8; S9, stage 9; S10, stage 10; S11, stage 11; S12, stage12; S13 stage13. *OsActin1* was used as an internal control. Each reaction had three biological repeats and error bars indicate the standard deviations (SD). **(B–E)** *In situ* hybridization of *OsCER1* in WT anthers. The anthers at stage 10 **(B)** and stage 11 **(D)** hybridized with an *OsCER1* antisense probe. The anthers at stage 10 **(C)** and stage 11 **(E)** hybridized with an *OsCER1* sense probe. Bp, bicellular pollen; Msp, microspore; T, tapetum. Bars = 50 μm in **(B–E)**.

To further characterize the expression pattern of *OsCER1*, we measured its transcript levels in different tissues by qRT-PCR. In rice, anther development can be divided into 14 stages based on morphological characteristics (Zhang et al., 2011). *OsCER1* was highly expressed in the panicles, particularly in the spikelets within anthers at stages 10 and 11, whereas its expression level was lower in the vegetative organs (**Figure 2A**). This finding indicates that *OsCER1* might be involved in panicle development in rice. To explore the spatiotemporal expression of *OsCER1* during anther development, RNA *in situ* hybridization of the WT anthers was performed. Rare *OsCER1* signals were detectable in anthers from stages 6 to 9 (**Supplementary Figure 1**), which refer to the stages from microspore mother cells generation to microspores generation. However, strong RNA *in situ* hybridization signals were detected in the tapetum cells with swollen structure at stage 10 (**Figure 2B**). Also, a high *OsCER1* expression level was detected in bicellular pollens undergoing the first mitosis at stage 11 (**Figure 2D**). In the negative control, hybridization with the *OsCER1* sense probe did not generate any detectable signals (**Figures 2C,E**). These results demonstrate that *OsCER1* is highly expressed in tapetum cells and bicellular pollens of anthers at stages 10 and 11, respectively.

### Defects in Anther Development in Plants Expressing an *OsCER1* Antisense Construct

To analyze the biological function of *OsCER1*, we constructed an *OsCER1* antisense vector containing a gene-specific *OsCER1* fragment driven by the native promoter and transformed this construct into Zhonghua 11 calli (Hiei et al., 1994; Li J. et al., 2011). A total of nine independent *OsCER1A* lines were obtained and identified by Southern blotting (**Supplementary Figure 2**). Furthermore, we obtained two transgenic homozygous lines through genetic screening, lines *OsCER1A* 3-6 and *OsCER1A* 9-1, which exhibited lower *OsCER1* transcript levels as determined by semi-quantitative RT-PCR (**Supplementary Figure 3**). These two lines were then used in the subsequent experiments, and the *OsCER1A* 3-6 plants showed the lowest *OsCER1* expression.

**FIGURE 3:**
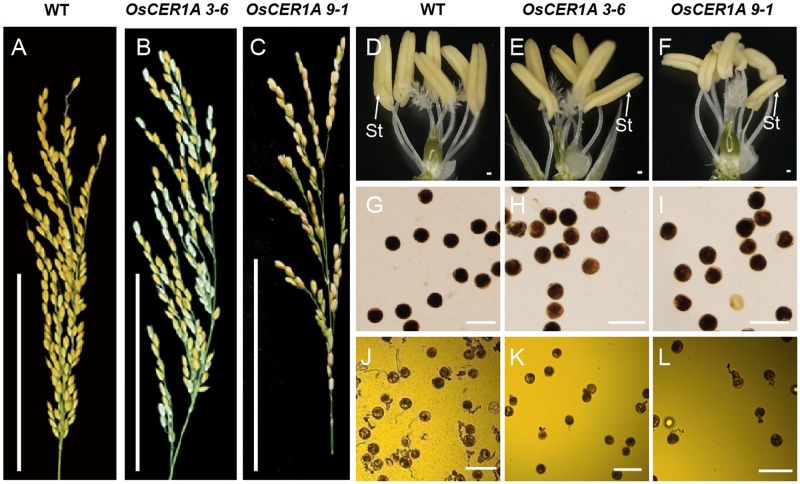
Comparison of the WT plants and *OsCER1A* lines. **(A–C)** Comparison of seed setting rate between the WT **(A)**, *OsCER1A* 3-6 **(B)**, and *OsCER1A* 9-1 **(C)** plants showing low seed setting rate in the *OsCER1A* plants. **(D–F)** A WT spikelet **(D)**, an *OsCER1A* 3-6 spikelet **(E)**, and an *OsCER1A* 9-1 spikelet **(F)** after removing the lemma and palea showing no obvious difference in stamens with each other. **(G)** Mature pollen grains stained with 1% I_2_/KI solution in the WT plants. **(H,I)** Abnormal pollen grains stained with 1% I_2_/KI solution in the *OsCER1A* 3-6 **(H)** and *OsCER1A* 9-1 **(I)** plants showing lightly stained pollen grains. **(J–L)** *In vitro* pollen germination of the WT **(J)**, *OsCER1A* 3-6 **(K)** and *OsCER1A* 9-1 **(L)** plants. St, stamen. Bars = 10 cm in **(A–C)**; 100 μm in **(D–F)**; 50 μm in **(G–L)**.

The plants of *OsCER1A* 3-6 and *OsCER1A* 9-1 at maturity stage were slightly smaller than the WT plants (**Supplementary Figure 4**). The most remarkable feature of the *OsCER1A* plants was the lower seed setting. The seed setting of the *OsCER1A* 3-6 plants only 50.14%, which is lower than that of the *OsCER1A* 9-1 plants (54.79%) and significantly reduced compared to that of the WT plants (94.20%) (**Figures 3A–C** and **Supplementary Table 1**). The anthers of the *OsCER1A* 3-6 and *OsCER1A* 9-1 plants also showed a normal stamen morphology (**Figures 3D–F**) and SEM showed slightly higher concentration of wax crystal in the surface of *OsCER1A* anthers and no obvious alteration in *OsCER1A* pollens morphology (**Supplementary Figure 5**). To explore the reason of sterility, we examined pollen viability and pollen tube growth by I_2_/KI staining and *in vitro* germination analysis, respectively. The pollen grains of the *OsCER1A* 3-6 and *OsCER1A* 9-1 showed lighter staining than that of the WT (**Figures 3G–I**), suggesting lower pollen viability in the *OsCER1A* plants. Additionally, statistical analysis of the *in vitro* germination demonstrated that none of the *OsCER1A* 3-6 and *OsCER1A* 9-1 pollens were able to germinate normal pollen-tubes compared to the 42.33% normal pollen-tube rate of the WT pollens germination (**Figures 3J–L**). These results strongly indicate that a decrease in *OsCER1* expression affects pollen fertility and pollen germination.

**FIGURE 4:**
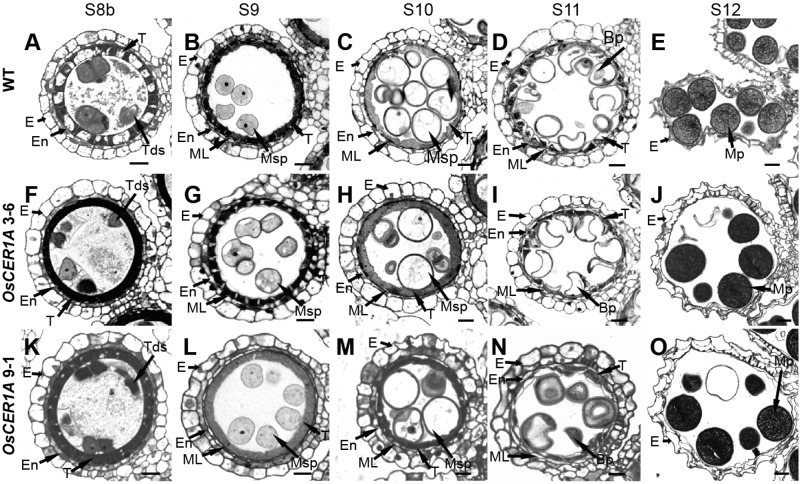
Comparison of transverse semi-thin sections of anthers from the WT plants and *OsCER1A* lines. **(A–E)** Transverse semi-thin sections of a single locule of the WT anthers at stage 8b (S8b) **(A)**, stage 9 (S9) **(B)**, stage 10 (S10) **(C)**, stage 11 (S11) **(D)**, and stage 12 (S12) **(E)**. **(F–J)** Transverse semi-thin sections of a single locule of the *OsCER1A* 3-6 anthers at S8b **(F)**, S9 **(G)**, S10 **(H)**, S11 **(I)**, and S12 **(J)**. **(K–O)** Transverse semi-thin sections of a single locule of the *OsCER1A* 9-1 anthers at S8b **(K)**, S9 **(L)**, S10 **(M)**, S11 **(N)**, and S12 **(O)**. Bp, bicellular pollen; E, epidermis; En, endothecium; ML, middle layer; MP, mature pollen; Msp, microspore; T, tapetum; Tds, tetrads. Bars = 20 μm.

To examine defects in anther development in the *OsCER1A* plants, we evaluated transverse semi-thin sections of the WT and *OsCER1A* anthers by light microscopy. At stage 8, the dyad cells underwent normal meiosis and produced tetrads, and tapetum displayed shrunk and vacuolated cytoplasm of the WT anthers (**Figure 4A**). However, the tetrads became abnormally vacuolated in the *OsCER1A* 3-6 and *OsCER1A* 9-1 anthers (**Figures 4F,K**), and tapetal cells exhibit non-vacuoles of the *OsCER1A* 3-6 anthers (**Figure 4F**) and small vacuoles of the *OsCER1A* 9-1 anthers (**Figure 4K**). At stage 9, new microspores were generated and released from the tetrads, and the vacuoles in the tapetal cells were reabsorbed and the cytoplasm appeared highly condensed with intense staining of the WT anthers (**Figure 4B**). In contrast, the thicker tapetum was observed in the *OsCER1A* 3-6 and *OsCER1A* 9-1 anthers at the same stage (**Figures 4G,L**). At stage 10, the microspores exhibited a round-shape within the enlarging vacuoles and the tapetum continued to degenerate, forming swollen and lighter stained tapetal cells in the WT anthers (**Figure 4C**), whereas darkly stained tapetal cells appeared in the *OsCER1A* 3-6 and *OsCER1A* 9-1 anthers at this stage (**Figures 4H,M**). At stage 11, the microspores underwent the first mitosis, and the tapetal layer continued to degenerate and formed large, clear vacuoles in the WT anthers (**Figure 4D**); the small vacuoles could be observed in the *OsCER1A* 9-1 tapetal cells (**Figure 4N**), whereas the tapetal cells still showed intense staining cytoplasm in the *OsCER1A* 3-6 anthers (**Figure 4I**). Subsequently, the mature pollen grains of the *OsCER1A* 3-6 and *OsCER1A* 9-1 plants were abnormally deeply stained compared with that of the WT plants (**Figures 4E,J,O**). These results suggest that the *OsCER1A* plants underwent abnormal tapetum and pollen development.

**FIGURE 5:**
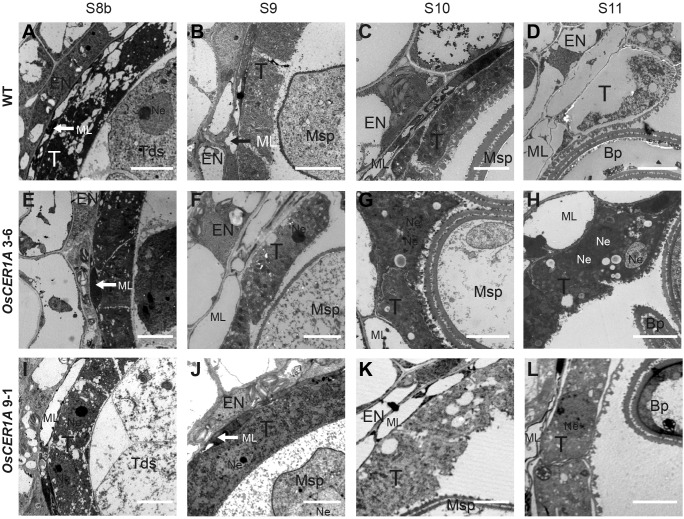
Comparison of transmission electron microscopy (TEM) of anthers from the WT plants and *OsCER1A* lines. **(A–D**) TEM analysis of the WT anthers at stage 8b (S8b) **(A)**, stage 9 (S9) **(B)**, stage 10 (S10) **(C)**, and stage 11 (S11) **(D)**. **(E–H)** TEM analysis of the *OsCER1A* 3-6 anthers at S8b **(E)**, S9 **(F)**, S10 **(G)**, and S11 **(H)**. **(I–L)** TEM analysis of the *OsCER1A* 9-1 anthers at S8b **(I)**, S9 **(J)**, S10 **(K)**, and S11 **(L)**. Bp, bicellular pollen; En, endothecium; ML, middle layer; Msp, microspore; Ne, nucleus; T, tapetum; Tds, tetrads. Bars = 10 μm.

To further characterize the details of the defect in the tapetal cells of the *OsCER1A* plants, the ultra-structural features of the tapetal cells were assessed by TEM. From stages 8b to 11, the tapetum of the WT plants showed signs of degeneration (**Figures 5A–D**). In contrast, the tapetal cells of the *OsCER1A* 3-6 and *OsCER1A* 9-1 plants showed the state of condensed cytoplasm had been maintained from stages 8b to 11 (**Figures 5E–L**). In particular, at stage 11, obviously large vacuoles and fragmented cytoplasm were observed in the tapetal cells of the WT anthers (**Figure 5D**). A densely stained cytoplasm full of the whole tapetal cells and a dense cytoplasm with small vacuoles within tapetal cells were observed in the *OsCER1A* 3-6 and *OsCER1A* 9-1 plants, respectively (**Figures 5H,L**). These findings suggest that the tapetal cells of the *OsCER1A* plants underwent abnormal degeneration compared to that in the WT plants.

### Delayed Programmed Cell Death (PCD) in Tapetum of *OsCER1A* Plants

**FIGURE 6:**
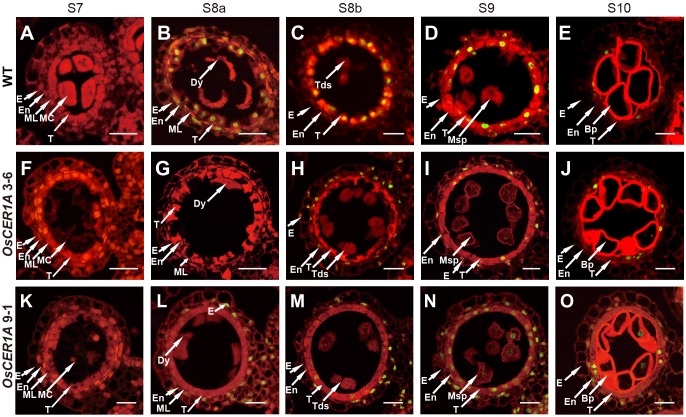
Programmed cell death (PCD) defects in the tapetal cells of the *OsCER1A* lines. **(A–E)** TUNEL assay of the WT anthers at stage 7 (S7) **(A)**, stage 8a (S8a) **(B)**, stage 8b (S8b) **(C)**, stage 9 (S9) **(D)**, and stage 10 (S10) **(E)**. **(F–J)** TUNEL assay of the *OsCER1A* 3-6 anthers at S7 **(F)**, S8a **(G)**, S8b **(H)**, S9 **(I)**, and S10 **(J)**. **(K–O)** TUNEL assay of the *OsCER1A* 9-1 anthers at S7 **(K)**, S8a **(L)**, S8b **(M)**, S9 **(N)**, and S10 **(O)**. The red fluorescence showed tissues stained with propidium iodide and the yellow fluorescence showed TUNEL-positive nuclei. Bp, bicellular pollen; Dy, dyad cell; E, epidermis; En, endothecium; MC, meiotic cell; ML, middle layer; Msp, microspore; Tds, tetrads; T, tapetum. Bars = 50 μm.

In rice, PCD is necessary for anther development, and abnormal PCD in tapetal cells may cause male sterility (Parish and Li, 2010; Qin et al., 2011; Shi et al., 2011; Tan et al., 2012). The hallmarks of PCD include DNA fragmentation, mitochondrial, and cytoskeletal disintegration, cellular condensation, and nuclear condensation (Parish and Li, 2010). The above TEM observations in the tapetum indicate that the tapetum degeneration in the *OsCER1A* plants was affected during anther development. To determine the effect of *OsCER1* downregulation on tapetum degeneration and PCD during anther development, we examined DNA fragmentation of the WT, *OsCER1A* 3-6 and *OsCER1A* 9-1 tapetum using the TUNEL assay. In the WT tapetal cells, TUNEL-positive signals were first detected at stage 8a (**Figures 6A,B**), and the signals were strongest at stage 8b (**Figure 6C**). Subsequently, the signals in the tapetal cells gradually weakened from stage 9 to stage 10 in the WT tapetal cells (**Figures 6D,E**). However, in the *OsCER1A* plants, no TUNEL signals were observed in the *OsCER1A* 3-6 and *OsCER1A* 9-1 tapetal cells at stages 7 (**Figures 6F,K**) and 8a (**Figures 6G,L**). At stage 8b, no apoptotic characteristics were detected in the *OsCER1A* 3-6 tapetal cells (**Figure 6H**), and weakly TUNEL signals were detected in the *OsCER1A* 9-1 tapetal cells (**Figure 6M**). Stronger but fewer TUNEL-positive signals and several intense TUNEL-positive signals were observed in the *OsCER1A* 3-6 and *OsCER1A* 9-1 tapetal cells at stage 9, respectively (**Figures 6I,N**). Until stage 10, signals in the *OsCER1A* 3-6 and *OsCER1A* 9-1 tapetal cells remained distinct (**Figures 6J,O**). These results suggest the initiation of tapetal PCD in the *OsCER1A* 3-6 and *OsCER1A* 9-1 tapetum was delayed from stage 8a to stage 9 and stage 8b, respectively. These results suggest that the abnormal non-vacuolization in the *OsCER1A* tapetum during stage 8b was coupled with delayed tapetal PCD may result in male sterility.

### Defects in Plastids During Anther Development in the *OsCER1A* Plants

**FIGURE 7:**
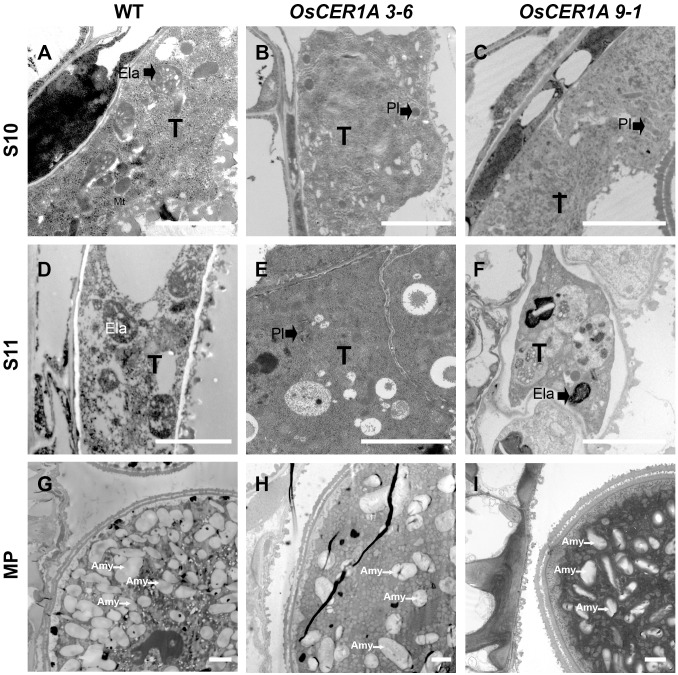
TEM of tapetum cells at stage 10 (S10), at stage 11 (S11) and mature pollen (MP) in the WT plants and *OsCER1A* lines. **(A–C)** Tapetal regions at S10 of the WT **(A)**, *OsCER1A* 3-6 **(B)**, and *OsCER1A* 9-1 **(C)** plants. **(D–F)** Tapetum regions at S11 of the WT **(D)**, *OsCER1A* 3-6 **(E)**, and *OsCER1A* 9-1 **(F)** plants. **(G–I)** Pollen cross sections of the WT **(G)**, *OsCER1A* 3-6 **(H)**, and *OsCER1A* 9-1 **(I)** plants. Amy, amyloplast; Ela, elaioplast; Pl, plastid; T, tapetum. Bars = 2 μm.

Abnormal tapetal elaioplasts were also detected in the *OsCER1A* plants at stage 11 (**Figures 5H,L**). TEM was used to observe tapetal cells and pollens at higher magnification, which exhibited significant differences in plastid structure in the *OsCER1A* anthers relative to that in the WT anthers (**Figure 7**). The proplastids in the WT tapetal cells at stage 10 were beginning to develop into larger elaioplasts (**Figure 7A**). In contrast, in the *OsCER1A* 3-6 and *OsCER1A* 9-1 tapetal cells the plastids were observed without elaioplast-like shape at this stage (**Figures 7B,C**). At stage 11 in the WT anthers, the tapetal cells had generated into cellular debris and contained the large completely differentiated elaioplasts (**Figure 7D**). However, at this stage the *OsCER1A* 3-6 tapetal cells remained filled with cytoplasm including plastids that lacked a distinct boundary and a typical elaioplast structure, and in the *OsCER1A* 9-1 plants, tapetal cytoplasm was still dense including elaioplasts with abnormal structure (**Figures 7E,F**). In the WT plants, mature pollens were filled with amyloplasts that differentiated from proplastids (**Figure 7G**). In contrast, in the *OsCER1A* 3-6 and *OsCER1A* 9-1 plants, fewer amyloplasts were observed within the mature pollen grains and most of them exhibited abnormal morphology (**Figures 7H,I**). Taken together, these results suggest that the downregulation of *OsCER1* disrupts the development of elaioplasts and amyloplasts within tapetal cells and in microspores, respectively.

### *OsCER1* Is Involved in VLC Alkanes Biosynthesis

**FIGURE 8:**
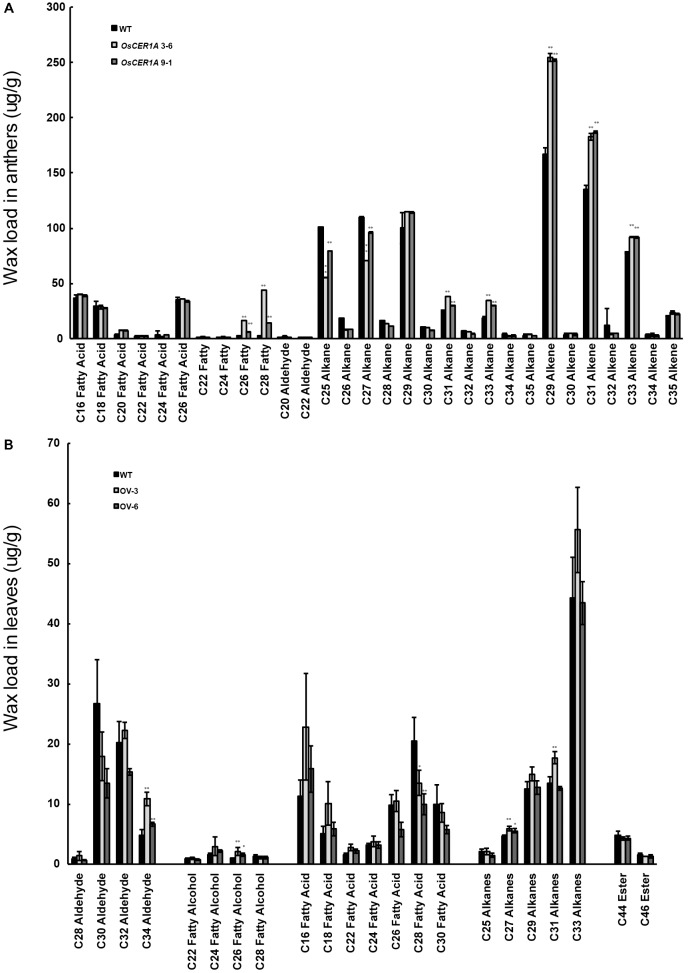
Analysis of wax constituents. **(A)** Wax constituents in whole anthers at stage 12 in the WT (Black bars), *OsCER1A* 3-6 (slightly gray bars) and *OsCER1A* 9-1 (darkly gray bars) plants. **(B)** Wax constituents in leaves in the WT (black bars), *OsCER1* overexpression 3 (OV-3) (slight gray bars) and *OsCER1* overexpression 6 (OV-6) (darkly gray bars) plants. Error bars indicate SD (*n* = 5). Statistical significance of differences between WT and transgenic plants means is indicated by ^∗^*p* < 0.05 and ^∗∗^*p* < 0.01.

In Arabidopsis, CER1 is involved in VLC alkanes biosynthesis (Bourdenx et al., 2011; Bernard et al., 2012). Our bioinformatics analysis showed that *OsCER1* possesses three conserved His-rich motifs and shares high sequence identity with CER1. The subcellular localization of the signal peptide of OsCER1 showed that the ER localization of the GFP fusion protein was detected by the co-localization of GFP fluorescence and KDEL-MCherry (ER marker) fluorescence (**Supplementary Figure 6**) (Zhou et al., 2015), which indicating that OsCER1 was localized in the ER. These results suggest that *OsCER1* may also be involved in the biosynthesis of VLC alkanes in rice. To investigate the alteration of wax profiles in the *OsCER1A* anthers, we measured the composition of chloroform-extractable cuticular wax in the mature anthers using gas chromatography-mass spectrometry (GC-MS). In the WT anthers, total wax content of dry, mature anthers was 999.63 μg/g, whereas in the *OsCER1A* lines, total wax increased by 16.2% to 1,161.64 μg/g in the *OsCER1A* 3-6 anthers and by 13.6% to 1135.81 μg/g in the *OsCER1A* 9-1 anthers (**Table 1**). Compared to the WT anthers, the alkene content in the *OsCER1A* 3-6 and *OsCER1A* 9-1 anthers was increased by 34.54% and by 43.18%, respectively (**Table 1**). The primary alcohol content was increased by 813.62% and by 218.41% in the *OsCER1A* 3-6 and *OsCER1A* 9-1 anthers, respectively (**Table 1**). However, alkane content decreased by 13.55% and 6.67% in the *OsCER1A* 3-6 and *OsCER1A* 9-1 anthers, respectively (**Table 1**). A significant reduction of alkanes C25 (45.26% and 21.27%) and C27 (35.46% and 12.38%) and a dramatic increase in corresponding primary alcohols C26 (573.26% and 146.1%) and C28 (2,494.40% and 477.79%) were detected in the *OsCER1A* 3-6 and *OsCER1A* 9-1 anthers, respectively (**Figure 8A** and **Supplementary Table 2**). These results indicate that *OsCER1* may function in alkane biosynthesis.

**Table 1:** Epicuticular wax composition of anthers from WT and *OsCER1A* lines.

Wax	Wild-type (μg/g)	*OsCER1A* 3-6 (μg/g)	*OsCER1A* 9-1 (μg/g)
			
	Mean ±*SD*	Mean ±*SD*	Mean ±*SD*
Fatty acids	110.34 ± 3.30	115.44 ± 2.00	113.42 ± 0.10
Primary alcohols	6.90 ± 0.84	63.04 ± 0.85	21.97 ± 0.05
Aldehydes	2.10 ± 0.05	2.56 ± 0.42	2.19 ± 0.002
Alkanes	413.95 ± 14.60	357.84 ± 0.62	386.32 ± 0.31
Alkenes	420.01 ± 14.60	565.10 ± 4.43	563.57 ± 0.49
Stigmasterol	46.33 ± 5.38	57.66 ± 2.59	48.33 ± 0.05
Total wax	999.63 ± 34.46	1161.64 ± 5.96	1135.81 ± 1.00


To confirm whether *OsCER1* is involved in VLC alkanes biosynthesis, we obtained 2 homozygous *OsCER1* overexpression lines, OV-3 and OV-6, which exhibited higher *OsCER1* transcript levels, as determined by qRT-PCR (**Supplementary Figure 7**). These two lines were then used in the subsequent wax composition analysis, and the *OsCER1* expression of OV-3 exhibited higher than that of OV-6. To avoid the effect of native *OsCER1*, we selected leaves that showed minimal native *OsCER1* expression for wax composition analysis. Compared to the WT leaves, the C27, C29, C31, C33 VLC alkanes increased in the OV-3 leaves, and C27 alkanes were increased in the OV-6 leaves (**Figure 8B**). The C27 alkanes were showed a significant increase (24.71% and 16.77%) in the OV-3 and OV-6 leaves, respectively (**Figure 8B** and **Supplementary Table 3**). We also detected a significant reduction of the corresponding fatty acid C28 (34.17% and 51.54%) in the OV-3 and OV-6 leaves, respectively (**Figure 8B** and **Supplementary Table 3**). The reduction in the content of VLC alkanes of the *OsCER1A* plants and the increase in the content of VLC alkanes of *OsCER1* overexpression plants suggest that *OsCER1* functions in VLC alkanes biosynthesis.

### Altered Gene Expression Affects Lipid Metabolism and Tapetum Development in *OsCER1A* Plants

**FIGURE 9:**
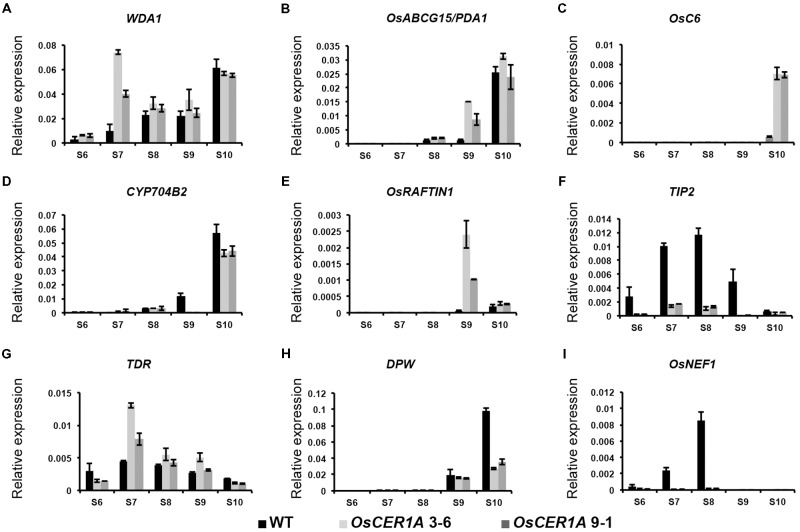
Expression analysis of genes related to lipid metabolism and pollen wall biosynthesis in the WT and *OsCER1A* lines by qRT-PCR. **(A–I)** Expression analysis of *WDA1* **(A)**, *OsABCG15/PDA1* **(B)**, *OsC6* **(C)**, *CYP704B2* **(D)**, *OsRAFTIN1* **(E)**, *TIP2* **(F)**, *TDR* **(G)**, *DPW* **(H)**, and *OsNEF1* **(I)** in the WT, *OsCER1A* 3-6 and *OsCER1A* 9-1 plants with anthers from stages 6 to 10. *OsActin1* was used as a control. Black bars represent the WT plants. Slightly gray bars represent the *OsCER1A* 3-6 plants. Darkly gray bars represent the *OsCER1A* 9-1. Each reaction had three biological repeats. Error bars indicate SD (*n* = 4). Gene names are abbreviated as follows: *DPW, Defective Pollen Wall*; *OsABCG15/PDA1, Rice ATP binding cassette subfamily G 15/ Post-meiotic deficient anther 1*; *OsNEF1, Rice No Exine Formation 1*; *TDR, Tapetum Degeneration Retardation*; *TIP2, TDR Interacting Protein 2*; *WDA1, Wax-deficient Anther 1*.

The reduced fertility and altered lipid profiles of the *OsCER1A* plants implied that some genes that were critical for anther development and lipid metabolism might be affected. Therefore, we used qRT-PCR to examine five genes involved in lipid metabolism and anther development, namely, *WDA1* (Jung et al., 2006; Islam et al., 2009), *OsABCG15/PDA1* (Qin et al., 2013; Zhu et al., 2013), *OsC6* (Zhang et al., 2010), *OsRAFTIN1* (Wang et al., 2003), and *CYP704B2* (Li et al., 2010) in the WT, *OsCER1A* 3-6, and *OsCER1A* 9-1 spikelets with anthers from stages 6 to 10. Among these genes, OsCER1 showed 63% identity with WDA1, which also belonged to the clade I (**Figure 1A**) in rice. The expression level of *WDA1*, which is essential for anther wall and pollen development, significantly increased in *OsCER1A* spikelets with anthers at stage 7 (**Figure 9A**). An increase in the expression of *OsABCG15/PDA1*, which encodes a lipid-soluble precursor transporter, was observed at stage 9 (**Figure 9B**), whereas an upregulation of *OsC6*, which is required for transport of lipophilic materials, was detected at stage 10 in the *OsCER1A* plants (**Figure 9C**). *CYP7042B2* and *OsRAFTIN1* are important for exine formation during pollen development. The expression pattern of *CYP704B2* was similar to that of WT plants from stages 6 to 8, but at stages 9 and 10, the expression levels of *CYP704B2* significantly decreased (**Figure 9D**). *OsRAFTIN1* expression increased at stages 9 and 10 in the *OsCER1A* plants (**Figure 9E**).

The abnormal differentiation of tapetal cells in the *OsCER1A* plants implied that some genes critical for tapetum degeneration could be affected. We analyzed two basic helix-loop-helix (bHLH) transcription factor genes, *TIP2* and *TDR* (Zhang et al., 2008; Yi et al., 2012, 2016; Fu et al., 2014). *TIP2* functions in tapetum differentiation during early anther development, and *TIP2* transcript levels significantly decreased from stage 6 to stage 10 in the *OsCER1A* plants (**Figure 9F**). *TDR* functions downstream of *TIP2*, and unexpectedly, *TDR* transcript levels significantly increased from stages 7 to 8 (**Figure 9G**). These results suggest that *OsCER1* is involved in the regulation of tapetum development probably not via the TIP2-TDR pathway.

Because plastids development in the *OsCER1A* plants was blocked, we analyzed two plastidial genes, *DPW* and *OsNEF1.* DPW is a plastid-localized fatty acid reductase that is involved in primary fatty alcohol synthesis and pollen wall formation (Shi et al., 2011). The expression of *DPW* started to decrease at stage 9 in the *OsCER1A* plants, suggesting that *DPW* is affected by the downregulation of *OsCER1* (**Figure 9H**). *OsNEF1* shares high identity (up to 60%) with Arabidopsis *NEF1*, which encodes a regulator of primexine formation that is localized in plastids (Ariizumi et al., 2004; Shi et al., 2011). The expression level of *OsNEF1* significantly decreased at stages 6, 7, and 8 (**Figure 9I**), indicating that the downregulation of *OsCER1* also results in a decrease in *OsNEF1* expression.

## Discussion

### *OsCER1* Is Associated With VLC Alkanes Biosynthesis

Cuticular wax coats the surface of anthers, and the disruption of anther wax synthesis is frequently accompanied by abnormal pollen development (Jung et al., 2006; Jessen et al., 2011; Leide et al., 2011; Smirnova et al., 2013; Haslam et al., 2015). VLC alkanes and other VLCFA derivatives are produced via the alkane-formation pathway (Greer et al., 2007; Bernard et al., 2012; Wang et al., 2015b). There was strong evidence for the involvement of Arabidopsis CER1 in VLC alkanes biosynthesis (Aarts et al., 1995; Bourdenx et al., 2011). Arabidopsis CER1 contains three His-rich motifs (HX_3_H, HX_2_HH, and HX_2_HH, in which X stands for any amino acid) that are critical for its function in alkane synthesis (Aarts et al., 1995; Shanklin and Cahoon, 1998; Bourdenx et al., 2011). CsCER1, a CER1 homolog in cucumber sharing 61% identity with CER1, also contains three His-rich motifs (Wang et al., 2015b). Overexpression of *CsCER1* resulted in significant changes in VLC alkanes production, indicating that *CsCER1* also plays a role in VLC alkanes biosynthesis (Wang et al., 2015b). However, genes involved in VLC alkane forming have not been reported in rice, and the VLC alkanes biosynthesis pathway has not been elucidated. The present study determined that full-length OsCER1 shares 57% sequence identity with CER1; OsCER1 also contains the three conserved His-rich motifs. Furthermore, the proportion of C25 and C27 alkanes decreased in the *OsCER1A* plants. Accordingly, the concentration of C27 alkanes increased in the *OsCER1* overexpression plants. These results suggest that the synthesis of some species of VLC alkanes decreased when *OsCER1* was knocked down, and increased when *OsCER1* was overexpressed. Based on these findings, we suggest that *OsCER1* is involved in VLC alkanes biosynthesis and may be mainly involved in C27 alkanes in rice.

### *OsCER1* Is Involved in a Distinctive Wax Synthesis Pathway

In the present study *OsCER1* deficiency was also associated with an increase in the concentration of total waxes, alkenes, and primary alcohols, and with a decrease in the concentration of alkanes. Mutants of genes homologous to *OsCER1* in rice, including *WSL2, OsGL1-2, OsGL1-3, WDA1*, and *OsGL1-6*, exhibited decreased total waxes, alkenes, primary alcohols, and alkanes (Jung et al., 2006; Qin et al., 2011; Mao et al., 2012; Zhou et al., 2013, 2015). Among these genes, WDA1 is sharing the highest identity (to 63%) with OsCER1. *WDA1* is involved in wax synthesis and pollen development in rice, and loss-of-function mutant *wda1* exhibits significant reduction in the contents of all wax components (fatty acids, alcohols, alkanes, and alkene) (Jung et al., 2006). However, in our *OsCER1A* plants, only the contents of C25 and C27 alkane significantly decreased, whereas contents of C31 and C33 alkanes markedly increased. There is a possibility that the antisense construct intended for non-specifically targets *OsCER1* and *WDA1*. If this were the case, then the decrease in VLC alkane content in *OsCER1A* plants could be an indirect effect of double knockdown of *OsCER1* and *WDA1*. To exclude such a possibility, qRT-PCR was performed using the spikelets with anthers from stages 6 to 10 of the WT and *OsCER1A* plants. A significant increase in the expression of *WDA1* was observed at stage 7, whereas no distinct changes were observed at other anther development stages (**Figure 9A**). These findings suggest that *OsCER1* may participate in a different wax synthesis pathway other than *WDA1* and other reported homologies in rice.

### *OsCER1* Is Required for Plastid Development in Anthers

Plastids play crucial roles for successful anther development in angiosperms with different morphologies at different developmental stages. In rice tapetum plastids begin as proplastids and differentiate into elaioplasts, which are involved in the biosynthesis of tapetal lipids; in turn, these contribute to lipidic pollen wall formation (Lévesque-Lemay et al., 2016). Amyloplasts are terminally differentiated plastids that serve as sites for starch synthesis and storage in mature pollen grains (Matsushima et al., 2014). However, the mechanisms of plastid development during anther development are not well understood. In the *OsCER1A* plants, we observed abnormal plastids differentiation and alterations in the structure of the plastid in tapetal cells. These results reveal that decreased *OsCER1* could affect plastid development. Furthermore, a significant decrease in the number of amyloplasts was observed in mature pollen grains of the *OsCER1A* plants. A recent study showed that VLCFAs play an essential role in plastid division, and reduced levels of VLCFAs lead to a decrease in the number of plastids (Nobusawa and Umeda, 2012). Wax analysis showed that *OsCER1* knockdown plants disrupt the homeostasis of VLCFAs and their derivatives, which in turn could cause alterations in plastid division and development, thereby affecting the quantity of amyloplasts in mature pollens, which in turn influences pollen viability.

### Plastid Functions in Anther Development

*De novo* FA synthesis occurs in plastids, and lipid metabolism also affects plastid development. Furthermore, plastid-related lipid metabolism in the tapetum can influence anther development through aliphatic compound synthesis and secretion. In Arabidopsis, fatty acid export 1 (FAX1) has been localized to the inner envelope of chloroplasts and is involved in the transport of plastid fatty acids; in addition, *fax1* mutants produced shorter siliques containing almost no seeds (Li et al., 2015). In *Brassica napus*, Tic40 is a membrane-anchored co-chaperone homolog localized in the inner membrane of chloroplast that is mainly expressed in tapetum cells and microsporocytes. The *tic40* mutant exhibits delayed tapetum PCD and abnormal tapetal cell expansion and division (Dun et al., 2011). Plastid genes could also affect anther development through sugar signaling, such as glyceraldehyde-3-phosphate dehydrogenase (GAPCp) was localized in plastid, and *gapcp1gapcp2* double mutant displayed the sterility in Arabidopsis (Muñoz-Bertomeu et al., 2010). However, the role of plastids in anther development remains elusive.

Tapetum PCD is a precise, genetically controlled cellular process that is regulated by several transcription factors. For instance, the altered expression of some bHLH transcription factors strongly influences tapetum PCD in rice; these bHLH transcription factors include *Undeveloped Tapetum 1* (*UDT1/bHLH164*), *TDR1, TIP2*, and *Eternal Tapetum 1* (*EAT1*/*DTD1*/*bHLH141*) (Li et al., 2006; Ji et al., 2013; Niu et al., 2013; Fu et al., 2014; Ko et al., 2014). In rice, several other genes independent of the ROS and bHLH pathways also regulate tapetum PCD, including *Persistent Tapetal Cell 1* (*PTC1*), *Apoptosis inhibitor 5* (*API5*), and *Microspore and Tapetum Regulator1* (*MTR1*) (Li H. et al., 2011, Li X. et al., 2011; Tan et al., 2012; Cao et al., 2015). In addition to the PCD-related genes, various other genes are involved in tapetum development and degeneration in rice such as *Histone Monoubiquitination* (*OsHUB1*/*OsHUB2*), *Defective Tapetum and Meiocytes 1* (*DTM1*), and *Anther Development F-box* (*ADF*) (Aarts et al., 1995; Jung et al., 2005; Yi et al., 2012; Li et al., 2015; Cao et al., 2015). These genes form an extensive network that controls tapetum differentiation and PCD. The present studies had shown a delayed tapetum degeneration with a downregulation of *TIP2* but an upregulation of *TDR* in the *OsCER1A* plants. However, our results showed the opposite alterations in the primary alcohols and alkenes in *OsCER1A* plants compared to *tdr* mutants (Li et al., 2006). These findings suggest that delayed tapetum degeneration in *OsCER1A* plants may not via the TIP2-TDR pathway. However, whether plastids are involved in the delayed tapetum degeneration in *OsCER1A* plants and the potential roles of plastids in the regulation of tapetum PCD remain elusive.

In the present study, the reduced fertility *OsCER1A* plants exhibited abnormal plastid development, and delayed tapetum PCD during anther development. Alterations in the expression levels of genes involved in lipid metabolism, and plastidial genes were observed in the *OsCER1A* plants. This suggests that OsCER1, a protein involved in VLC alkanes biosynthesis, possibly generates some lipidic signals that impact genes involved in plastids development and tapetum PCD then affects anther development in rice.

## Author Contributions

CZ and HZ designed the project and wrote the manuscript. EN, LZ, JL, DJ, ZW, SZ, HQ, YZ, CW, SX, and ZL performed the experiments and analyzed the data.

## Conflict of Interest Statement

The authors declare that the research was conducted in the absence of any commercial or financial relationships that could be construed as a potential conflict of interest.

## Footnotes

1 http://www.ebi.ac.uk/Tools/msa/clustalo/

2 http://www.megasoftware.net/index.html

3 http://www.genome.jp/tools/clustalw/
