# Supplementary material for: OsCER1 Plays a Pivotal Role in Very-Long-Chain Alkane Biosynthesis and Affects Plastid Development and Programmed Cell Death of Tapetum in Rice (Oryza sativa L.)
Source: Front Plant Sci. 2018 Sep 6;9:1217. doi: 10.3389/fpls.2018.01217 (PMC6136457; doi:10.3389/fpls.2018.01217)
Supplement: Supplementary file 1 [file Table_1.doc]

**Supplementary Table 1.**Comparison of Seed Setting Rate.

| Line | Seed Setting rate (%) |
| --- | --- |
| WT | 94.20±3.84 |
| *OsCER1A* 3-6 | 50.14±14.25 |
| *OsCER1A* 9-1 | 54.79±15.71 |

**Supplementary Table 2.** Wax Composition of Rice Anthers.

| Wax contents | Composition | WT (μg/g) | *OsCER1A* 3-6 (μg/g) | *OsCER1A* 9-1 (μg/g) |
| --- | --- | --- | --- | --- |
|  |  | (Mean ±SD) | (Mean ±SD) | (Mean ±SD) |
| Fatty acid | C16:0 | 36.456  ±2.886 | 39.989  ±0.50 | 38.808  ±0.890 |
|  | C18:0 | 29.315  ±4.660 | 28.369  ±1.563 | 27.710  ±0.399 |
|  | C20:0 | 3.730  ±0.669 | 7.429  ±0.087 | 7.308  ±0.147 |
|  | C22:0 | 2.044  ±0.326 | 2.279  ±0.053 | 2.251  ±0.187 |
|  | C24:0 | 3.620  ±3.415 | 1.469  ±0.199 | 3.549  ±0.100 |
|  | C26:0 | 35.175  ±2.168 | 35.901  ±0.268 | 33.796  ±0.841 |
| Fatty alcohol | C22 | 0.704  ±0.649 | 1.641  ±0.478 | 0.876  ±0.130 |
|  | C24 | 1.326  ±0.082 | 1.287  ±0.304 | 0.940  ±0.261 |
|  | C26 | 2.408  ±0.148 | 16.212  ±0.117 | 5.926  ±0.057 |
|  | C28 | 2.463±  0.060 | 63.900  ±0.102 | 14.231  ±0.118 |
| Aldehyde | C20 | 1.131  ±0.099 | 1.862  ±0.557 | 1.213  ±0.130 |
|  | C22 | 0.969  ±0.118 | 0.703  ±0.137 | 0.980  ±0.238 |
| Alkane | C25 | 100.900  ±0.458 | 55.229  ±0.120 | 79.439  ±0.167 |
|  | C26 | 18.385  ±0.196 | 8.070  ±0.202 | 8.463  ±0.108 |
|  | C27 | 109.446  ±0.772 | 70.636  ±0.224 | 95.901  ±0.491 |
|  | C28 | 16.522  ±0.238 | 13.678  ±0.007 | 11.340  ±0.082 |
|  | C29 | 100.084  ±14.062 | 114.944  ±0.161 | 114.122  ±0.578 |
|  | C30 | 10.249  ±0.206 | 10.102  ±0.043 | 7.359  ±0.089 |
|  | C31 | 25.786  ±0.278 | 38.245  ±0，049 | 29.947  ±0.321 |
|  | C32 | 7.249  ±0.055 | 6.117  ±0.212 | 4.430  ±0.129 |
|  | C33 | 18.398  ±1.314 | 34.779  ±0.069 | 29.934  ±0.188 |
|  | C34 | 4.335  ±0.400 | 2.086  ±0.300 | 2.823  ±0.834 |
|  | C35 | 2.596  ±1.507 | 3.952  ±0.321 | 2.592  ±0.256 |
| Alkene | C29 | 167.303  ±5.306 | 254.749  ±3.736 | 251.968  ±0.853 |
|  | C30 | 3.348  ±1.271 | 4.939  ±0.143 | 3.788  ±1.114 |
|  | C31 | 135.032  ±3.451 | 182.641  ±3.018 | 186.811  ±0.848 |
|  | C32 | 12.239  ±14.939 | 4.138  ±0.435 | 4.629  ±0.036 |
|  | C33 | 78.091  ±0.877 | 91.994  ±0.398 | 91.426  ±0.658 |
|  | C34 | 3.663  ±0.179 | 3.302  ±1.596 | 2.669  ±0.712 |
|  | C35 | 20.338  ±0.534 | 23.334  ±1.461 | 22.276  ±0.593 |

**Supplementary Table 3. Wax Composition of Rice Leaves.**

| Wax contents | Composition | WT (μg/g) | OV-3 (μg/g) | OV-6 (μg/g) |
| --- | --- | --- | --- | --- |
|  |  | (Mean ±SD) | (Mean ±SD) | (Mean ±SD) |
| Fatty acid | C16:0 | 11.311  ±2.759 | 22.853  ±8.855 | 15.841  ±3.864 |
|  | C18:0 | 5.034  ±1.246 | 10.111  ±3.614 | 5.856  ±1.134 |
|  | C22:0 | 1.507  ±0.261 | 2.811  ±0.595 | 2.172  ±0.368 |
|  | C24:0 | 3.176  ±0.335 | 3.791  ±0.923 | 3.179  ±0.531 |
|  | C26:0 | 9.788  ±1.813 | 10.534  ±1.780 | 5.783  ±1.223 |
|  | C28:0 | 20.519  ±3.837 | 13.507  ±2.065 | 9.943  ±1.706 |
|  | C30:0 | 10.003  ±3.164 | 8.560  ±1.580 | 5.707  ±0.679 |
| Fatty alcohol | C22 | 0.851  ±0.236 | 0.969  ±0.193 | 0.729  ±0.092 |
|  | C24 | 1.516  ±0.305 | 2.891  ±1.541 | 2.185  ±0.211 |
|  | C26 | 1.005  ±0.048 | 2.101  ±0.655 | 1.555  ±0.254 |
|  | C28 | 1.313  ±0.208 | 1.100  ±0.229 | 1.115  ±0.242 |
| Aldehyde | C28 | 0.930  ±0.209 | 1.364  ±0.749 | 0.666  ±0.027 |
|  | C30 | 26.671  ±7.326 | 17.950  ±4.060 | 13.446  ±2.470 |
|  | C32 | 20.205  ±3.553 | 22.232  ±1.377 | 15.298  ±0.534 |
|  | C34 | 4.866  ±4.866 | 10.864  ±1.147 | 6.664  ±0.363 |
| Alkane | C25 | 2.125  ±0.424 | 2.064  ±0.565 | 1.490  ±0.394 |
|  | C27 | 4.741  ±0.331 | 5.913  ±0.374 | 5.536  ±0.435 |
|  | C29 | 12.555  ±1.189 | 14.985  ±1.124 | 12.739  ±1.126 |
|  | C31 | 13.501  ±1.019 | 17.711  ±1.049 | 12.644  ±0.339 |
|  | C33 | 44.339  ±6.674 | 55.601  ±7.076 | 43.456  ±3.577 |
| Ester | C44 | 4.766  ±0.742 | 4.251  ±0.273 | 4.326  ±0.319 |
|  | C46 | 1.573  ±1.573 | 1.294  ±0.022 | 1.264  ±0.266 |

**Supplementary Table 4.** Primers Used in This Study.

| **Primer name** | **Sequence (5’ – 3’)** |
| --- | --- |
| *OsCER1* q-pcr F | CTTCATCATACGCAGTTCC |
| *OsCER1* q-pcr R | TGTTTCTTCCGTTCCTTTC |
| *OsCER1* HIS F | CTCTGGAAGGATGGAACATG |
| *OsCER1* HIS R | AGTTCGTTTGCACTGTGCCTC |
| *OsCER1*-A F | GGATCCATCTGATTTTGCTGTGTTGC |
| *OsCER1*-A R | GTCGACATGGTCAGCTGCCATTCG |
| *OsCER1-*P F | GAATTCAGATGAACACCGCCAATGAG |
| *OsCER1-*P R | GGATCCTGTGGTCACTCTGCCTCAC |
| *OsCER1-eGFP F* | GAATTCATGGCGACCAAACCGGGC |
| *OsCER1-eGFP R* | GGATCCTGGCCCCCGTGGTGGCGCA |
| *OsCER1-*OE F | GAATTCATGGCGACCAAACCGGGC |
| *OsCER1*-OE R | GAATTCAGCTTTAGTGAGAGGAAG |
| *OsActin1* F | GACATTCAGCGTTCCAGCCATGTAT |
| *OsActin1* R | TGGAGCTTCCATGCCGATGAGAGAA |
| *qPCR-OsActin1* F | CACATTCCAGCAGATGTGGA |
| *qPCR-OsActin1* R | GCGATAACAGCTCCTCTTGG |
| *Wda1* q-pcr F1 | CACTTTCTCTACACCCACT |
| *Wda1* q-pcr R1 | TATGGAAGCAGTTCCAGTC |
| *DPW* q-pcr F1 | GATGAGTTCATGCGTGAACC |
| *DPW* q-pcr R1 | AAGCACGCTTCTGCCATGAC |
| *OsABCG* q-pcr F1 | CAGTGTTGAGGTTGACATG |
| *OsABCG* q-pcr R1 | CAGCTGTTAGTGGGTTCTT |
| *CYP704B2* q-pcr F1  *CYP704B2* q-pcr R1  *OsNEF1* q-pcr F1 | GGCAGAGTTGTAGACATGCA  CGACAGTATGTCGTGCTTGA  TTCCTTCCACTTCCAATGATT |
| *OsNEF1* q-pcr R1 | GGAGTGACAGCGAGTAGCAA |
| *OsRAFTIN1* q-pcr F1 | CCTTTCGGGTACGACTACAAG |
| *OsRAFTIN1* q-pcr R1 | CTACGCCGTCGAGCTCTTCA |
| *OsC6* q-pcr F1 | ATGGCGCCGTCCAAGTCCA |
| *OsC6* q-pcr R1 | AAGAGTAGAGAGGCGGCACA |
| *TDR* q-pcr F1 | GGAGGAGTAACAAGGACCCA |
| *TDR* q-pcr R1 | ACCTCCAGCAGCGAGTCCCT |
| *TIP2* q-pcr F1 | CTTCATGGTCGGACCCTTCG |
| *TIP2* q-pcr R1 | CGTCCACGAACGTCTCCTTG |
| *Hpt* Southern F | *TCCGGAGCCTCCGCTCGAAGTAG* |
| *Hpt* Southern R | *CTGAACTCACCGCGACGTCTGTC* |

Supplementary Data Set 1.

| **gene name** | **protein sequence** |
| --- | --- |
| CER3 | MVASAWWNGNKYYAAAVVYSWVYDSKVWCHCGKAVHWSVNNMVTRTRNKGDKDHWHWDNYAVSCYMSMMMNSWNTKGAVHVTSYYHRSHRNNYTHYHSHHSSVHMTAGNATNCVVAGVGCCGVGSSAYGYAVMDMRCGHCNVSHKVRYYTTYHSHHMGTNCMDVGDTNNSWKKRSAGRKRVVAHGVDVMSAMHAVRSASMYTTRMWTCVMGMWAWSKTSYTRNNCTWGVRGYATKGNDAARADKGVKVSAANKNANGGGTVNKHDRVRVVHGNTTAAVYKDVNVTGATSKGRAAYCRRGVRVMTSMRKKAVNNVVTKYNAAHCKTWVGKWTRSWAAGTHHVVKRRNCTYGDAAMKKDVGGTCYTMRGVVHACHAGGVVHMGWKHHVGADVDRDVWAAMKYGSAVSSTN |
| BnCER1 | MATKPGILTDWPWTPLGNFKYIVIAPWAVHSTYKFVTDDPVDLGYSLVLPFLLFRILHNQVWISLSRYYTTKGKRRILDKGIDFNQVDRETNWDDQILFNGLLFYIGIMLLPQAKQLPWWRTDGVLMAAMLHAGPVEFLYYWLHKALHHHFLYSRYHSHHHSSIVTEPITSVIHPFAEHIAYFILFAIPLLTTLLTKTASIASFSGYVIYIDFMNNMGHCNFELVPKRLFHLFPPLKYLCYTPSFHSLHHTQFRTNYSLFMPLYDYIYGTMDETSDTLYEKSLERGEDRVDVVHLTHLTTPESIYHLRIGLASFASYPFSYRWFMRLLWPFTSLSMLFTLFYASLFVSERNSFEKLNLQSWIIPRYNLQYLLKWRKDAINNMIEKAILEANEKGVKVLSLGLMNQGEELNRNGEVYIHKHPEMKVRVVDGSRLTAAVVINSLPKSTTKIVMTGNLTKVAYTIASALCQRGVEVLTLLPEEYEKLSSFVPKECRDRLILLTSETLASNKVWLMGEGTTREEQEMATKGTLFIPFSQFPLKQLRRDCIYHTPPALIIPKSLVNIHSCENWLPRKAMSATRVAGILHALEGWETHECGTSNILLSDLDQVWEACLSHGFQPLLLPHHFQYP |
| CsCER1 | MASKPGILTDWPWKPLGSFKFVILTPWVIHSSYLYFKGGEKRDLSYILIFPFLVLRMIHNQIWISLSRYQTAKGTKRIVDKPIEFEQVDRESSWDDQILFNGLLFCLGRMVVEKGENLPLWRTNGVVIAALLHAGPVEFLYYWFHRALHHHFLYSRYHSHHHSSIATEPITSVIHPFAEHIVYFLLFTIPLLVTVLTETASIGSFVLYVMFIDFMNNMGHCNFEIVPKSLFFIFPPLKYLIYTPSFHSLHHTQFRTNYSLFMPIYDYIYGTVDKNSDSLYENSLLREEEVADVVHLSHLTTPQSIYHMRLGLATVASQPFTSKWWLTLLWPFTSFYVLATSFYGHIFVYERNTFKALKLQSWVIPRFNLQYFMKGRREAINKLIEAAILDADKKGVKVLSLGLLNQGKELNEYGEFYIHKYPNLRIKLVDGSSLAAAIVINTIPKATTKVLLRGNLSKVAYAIADALCQLGFQVATLYENEHKKLKSKVTTNSNNLVLAKITTHKIWIVGDGLEEFEQLNAPKGTIFIPYSQFPPQRLRKDCYYHITPSMRVPSSFQNIDSCENWLPRRVMSAWRMAGILHALEGREGHECGETMLSLDDAWRASLENGFLPLEIPSI |
| CER1 | MATKPGVLTDWPWTPLGSFKYIVIAPWAVHSTYRFVTDDPEKRDLGYFLVFPFLLFRILHNQVWISLSRYYTSSGKRRIVDKGIDFNQVDRETNWDDQILFNGVLFYIGINLLPEAKQLPWWRTDGVLMAALIHTGPVEFLYYWLHKALHHHFLYSRYHSHHHSSIVTEPITSVIHPFAEHIAYFILFAIPLLTTLLTKTASIISFAGYIIYIDFMNNMGHCNFELIPKRLFHLFPPLKFLCYTPSYHSLHHTQFRTNYSLFMPLYDYIYGTMDESTDTLYEKTLERGDDIVDVVHLTHLTTPESIYHLRIGLASFASYPFAYRWFMRLLWPFTSLSMIFTLFYARLFVAERNSFNKLNLQSWVIPRYNLQYLLKWRKEAINNMIEKAILEADKKGVKVLSLGLMNQGEELNRNGEVYIHNHPDMKVRLVDGSRLAAAVVINSVPKATTSVVMTGNLTKVAYTIASALCQRGVQVSTLRLDEYEKIRSCVPQECRDHLVYLTSEALSSNKVWLVGEGTTREEQEKATKGTLFIPFSQFPLKQLRRDCIYHTTPALIVPKSLVNVHSCENWLPRKAMSATRVAGILHALEGWEMHECGTSLLLSDLDQVWEACLSHGFQPLLLPHH |
| GLOSSY1 | MGAALLASWPWDNLGLYKYVLYGPLVGKAVASRAWEAASPDRWILLLLLLFGLRALTYQLWSSFSNMLFATRRRRVVRDGVDFDQIDKEWDWDNFLILHALMAAAALCAFPSLRHLPAWDGRGFAVALVAHAAATEPLSYLAHRALHGSSGRLYARYHSLHHSSRVPQPFTAGLATPLEHVALGALMSLPLAAARAAGCASVALAFAYVLAFDSLRAMGHCNVEVVPASLFRAIPALRYVLYTPTYHAIHHTKKEANFCLFMPLFDLLGGTIDRRSWDMQRKMSAGVDEVPDFVFLAHVVDVMQSLHVPFVMRTFASTPFSVQLFLLPMWPFAFLVMLAMWVWSKTFVISCYNLRGRLHQIWAVPRYGFQYFLPFAKDGINRQIELAILRADKMGVKVLSLAALNKNEALNGGGTLFVNKHPDLRVRVVHGNTLTAAVILNEIPKGTAEVFLTGATSKLGRAIALYLCKKRVRVMMMTLSTERFQKIQKEAPAEFQQYLVQVTKYRSAQHCRTWIVGKWLSPREQRWAPPGTHFHQFVVPPIIGFRRDCTYGKLAAMRLPKDVRGLGACEYSLERGLVHACHAGGVVHFLEGYTHHEVGAIDVDRIDVVWEAALKHGLRPA |
| WSL2 | MGAAFLSSWPWDNLGAYKYVLYAPLVGKAVAGRAWERASPDHWLLLLLVLFGVRALTYQLWSSFSNMLFATRRRRIVRDGVDFGQIDREWDWDNFLILQVHMAAAAFYAFPSLRHLPLWDARGLAVAALLHVAATEPLFYAAHRAFHRGHLFSCYHLQHHSAKVPQPFTAGFATPLEQLVLGALMAVPLAAACAAGHGSVALAFAYVLGFDNLRAMGHCNVEVFPGGLFQSLPVLKYLIYTPTYHTIHHTKEDANFCLFMPLFDLIGGTLDAQSWEMQKKTSAGVDEVPEFVFLAHVVDVMQSLHVPFVLRTFASTPFSVQPFLLPMWPFAFLVMLMMWAWSKTFVISCYRLRGRLHQMWAVPRYGFHYFLPFAKDGINNQIELAILRADKMGAKVVSLAALNKNEALNGGGTLFVNKHPGLRVRVVHGNTLTAAVILNEIPQGTTEVFMTGATSKLGRAIALYLCRKKVRVMMMTLSTERFQKIQREATPEHQQYLVQVTKYRSAQHCKTWIVGKWLSPREQRWAPPGTHFHQFVVPPIIGFRRDCTYGKLAAMRLPKDVQGLGACEYSLERGVVHACHAGGVVHFLEGYTHHEVGAIDVDRIDVVWEAALRHGLRPV |
| OsGL1-2 | MAAPPLSSWPWASLGSYKYVLYGAVVWKVAEEWRQQGAAPVGSWWLHLLLLFAARGLTYQFWFSYGNMLFFTRRRRVVPDSVDFRQVDAEWDWDNFLLLQTLIGATLVGSPAVARQQLLLPSLKQAWDPRGWAIALLLHVLVAEPLFYWAHRALHRAPLFSRYHAAHHHASVTTPLTAGFGTPLESLLLTVVIGVPLAGAFLMGVGSVGLVYGHVLLFDFLRSMGYSNVEVISPRVFQAVPLLRYLIYTPTYLSLHHREKDSNFCLFMPIFDLLGGTLNHKSWELQKEVYLGKNDQAPDFVFLAHVVDIMASMHVPFVLRSCSSTPFANHFVLLPFWPVAFGFMLLMWCCSKTFLVSSYRLRGNLHQMWTVPRYGFQYFIPAAKKGINEQIELAILRADRMGVKVLSLAALNKNEALNGGGTLFVNKHPELRVRVVHGNTLTAAVILNEIPSNVKDVFLTGATSKLGRAIALYLCRKKIRVLMLTLSSERFLKIQREAPAEFQQYLVQVTKYQPAQNCKTWLVGKWLSPREQRWAPAGTHFHQFVVPPIIGFRRDCTYGKLAAMRLPKDVQGLGYCEYTMERGVVHACHAGGVVHFLEGWEHHEVGAIDVDRIDVVWKAALKHGLTPA |
| OsGL1-3 | MAISMASPLSSWPWAFLGSYKYLLYGPVVGKVVQEWREQGRLPLGTSWCLHLILLLALRSLTYQLWFSYGNMLFFTRRRRVVDDGVDFRQIDTEWDWDNMVIMQTLIAAVLVTSRVFPATSDLSAWDLRGWAIAVVLHVAVSEPAFYWAHRALHLGPLFSRYHSLHHSFQATQALTAGFVTPLESLILTLVAWAPLAGAFMAGHGAVSLVYGHILLFDYLRSMGYSNVEVISHKTFQDFPFLRYLIYTPSYLSLHHREKDSNFCLFMPLFDALGGTLNPKSWQLQKEVDLGKNHRVPDFVFLVHVVDVVSSMHVPFAFRACSSLPFATHLVLLPLWPIAFGFMLLQWFCSKTFTVSFYKLRGFLHQTWSVPRYGFQYFIPSAKKGINEMIELAILRADKMGVKVLSLAALNKNEALNGGGTLFVRKHPDLRVRVVHGNTLTAAVILNEIPGDVAEVFLTGATSKLGRAIALYLCRKKIRVLMLTLSTERFMNIQREAPAEFQQYLVQVTKYQAAQNCKTWIVGKWLSPREQRWAPAGTHFHQFVVPPIIGFRRDCTYGKLAAMRLPEDVEGLGTCEYTMGRGVVHACHAGGVVHFLEGWDHHEVGAIDVDRIDAVWNAALRHGLTPA |
| OsCER1 | MATRPGPLTEWPWHRLGNFKYVVMAPVVAHGARRVMRNGWGDLDIAFSLILPSLLLRMIHNQIWISLSRYQTARSKHRIVDRGIEFGQVDRERGWDDQILFNGLVFYAGYLAMPSVRRMPVWRTDGAVVTALVHTGPVEFLYYWFHRALHHHFLYSRYHSHHHASIVTEPITSVIHPFAEHVVYFILFAIPILSTIYLGNVSAMGIVGYIAYIDFMNNMGHCNFELVPEWIFQIFPPLKYLIYTPSFHSLHHTQFRTNYSLFMPFYDYIYNTMDKSSDELYESSLKGTEETPDLVHLTHMTNLQSAYHLRIGIASIASKPYSDSAWYMWTLWPLAWLSMVLAWIYGSSAFVVERIKLNKMKMQTWALPRYNFQYGLTWEREPINDLIEKAILDADMKGVKVISLGLLNQAKQLNGNGELFRQKYPKLGVRIIDGSGLATAVVLKSIPSDAKKVFLRTGTSKIARAIAIALCDRGVQVIMNEKEVYHMLKSQIPENRASYLKLSSDNVPQLWIVHNIDDNEQKMAPKGTIFIPISQFPLKKLRKDCTYMSTPAMRIPEEMKNIHSCENWLPRRVMSAWHIAGILHALEGWNMHECGDEMMDIEKSWSAAIRHGFLPLTKA |
| OsGL1-6 | MASKPGPLTQWPWHNLGNYKYALVAPSAAYSTYRFVTASSAAERDLLNFMVFPMLLLRLLYGQLWITVSRHQTARSKHKIVNKSLDFEQIDRERNWDDQIILTALVFYLVSATMPQAQVAPWWSTKGMVVTAVLHAGPVEFLYYWLHRALHHHWLYARYHSHHHASIVTEPITSVIHPFAEEVVYFVLLAIPILSTVATGTVSVVTANGYLVYIDFMNYLGHCNFELVPKCLFHVFPPLKYLLYTPSFHSLHHTQFRTNYSLFMPVYDYIYGTTDKSSDELYERTLQGRDEAAWRPDVVHLTHLTTPESVFHNRLGFAAVASNPLGAAASGHLLRAASAVASPLLSLFASTFRSEANRLDKLNIETWVIPRFTSHYTSKSDGYKVSRLIEKAVSDAEASGARVLTLGLLNQGYDLNRNGELYVVRKPSLKTKIVDGTSLAVAAVLNMIPQGTKDVLLLGNANKISLVLTLSLCKREIQVRMVNKELYECLKQQLQPEMQEHLVLSCSYSSKVWLVGDGVTDEEQMKAQKGSHFVPYSQFPPNKARNDCVYHCTPALLVPESFENLHVCENWLPRRVMSAWRAAGIVHALEKWDGHECGGRVTGVQKAWSAALARGFRPYDDHHHPGITHDGRGGL |
| WDA1 | MATNPGLFTEWPWKKLGSFKYVLLAPWVAHGWYEVATKGWREVDLGYIAILPSLLLRMLHNQAWITISRLQNARGRRQIVRRGIEFDQVDRERNWDDQIILSGILLYLGALYVPGGQHLPLWRTDGAGLIALLHAGPVEFLYYWFHRALHHHFLYTHYHSHHHSSIVTEPITSVIHPFAELVAYELLFSIPLIACALTGTASIIAFEMYLIYIDFMNNMGHCNFELVPSWLFTWFPPLKYLMYTPSFHSLHHTQFRTNYSLFMPFYDYIYNTMDKSSDTLYENSLKNNEEEEAVDVVHLTHLTTLHSIYHMRPGFAEFASRPYVSRWYMRMMWPLSWLSMVLTWTYGSSFTVERNVMKKIRMQSWAIPRYSFHYGLDWEKEAINDLIEKAVCEADKNGAKVVSLGLLNQAHTLNKSGEQYLLKYPKLGARIVDGTSLAAAVVVNSIPQGTDQVILAGNVSKVARAVAQALCKKNIKVTMTNKQDYHLLKPEIPETVADNLSFSKTGTAKVWLIGDGLDSAEQFRAQKGTLFIPYSQFPPKMVRKDSCSYSTTPAMAVPKTLQNVHSCENWLPRRVMSAWRIAGILHALEGWNEHECGDKVLDMDKVWSAAIMHGFCPVAQG |
