# Supplementary material for: OsCER1 Plays a Pivotal Role in Very-Long-Chain Alkane Biosynthesis and Affects Plastid Development and Programmed Cell Death of Tapetum in Rice (Oryza sativa L.)
Source: Front Plant Sci. 2018 Sep 6;9:1217. doi: 10.3389/fpls.2018.01217 (PMC6136457; doi:10.3389/fpls.2018.01217)
Supplement: Supplementary file 2 [file Image_1.pdf]

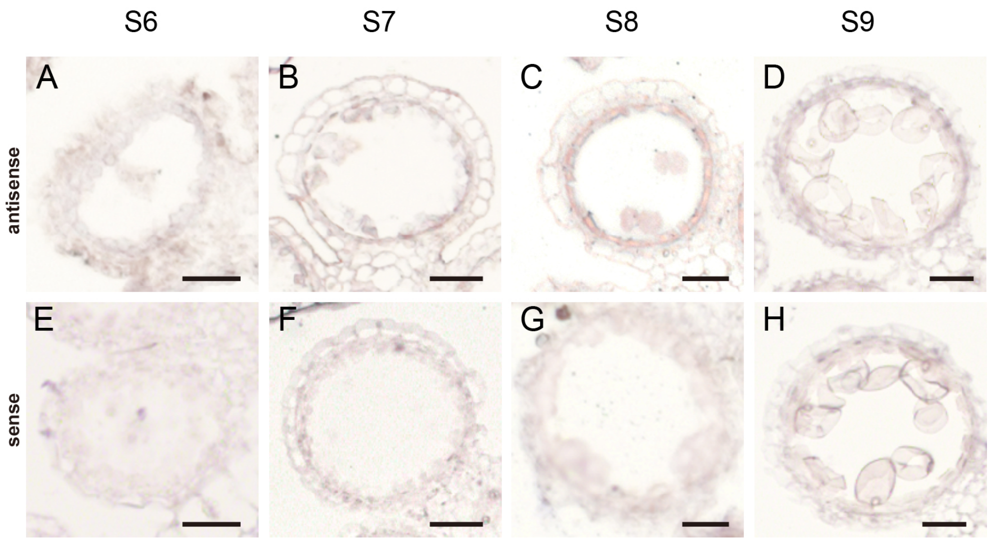

Supplementary Figure 1. *In situ* analysis of *OsCER1* in anthers from stage 6 to stage 9.

(A) to **(D)** *In situ* analysis of *OsCER1* mRNA in anthers from stages 6 to 9 with the *OsCER1* antisense probe. **(E)** to **(H)** *In situ* analysis of *OsCER1* mRNA in anthers from stages 6 to 9 with the *OsCER1* sense probe. Bars = 50 µm.
