# Supplementary material for: OsCER1 Plays a Pivotal Role in Very-Long-Chain Alkane Biosynthesis and Affects Plastid Development and Programmed Cell Death of Tapetum in Rice (Oryza sativa L.)
Source: Front Plant Sci. 2018 Sep 6;9:1217. doi: 10.3389/fpls.2018.01217 (PMC6136457; doi:10.3389/fpls.2018.01217)
Supplement: Supplementary file 3 [file Image_2.pdf]

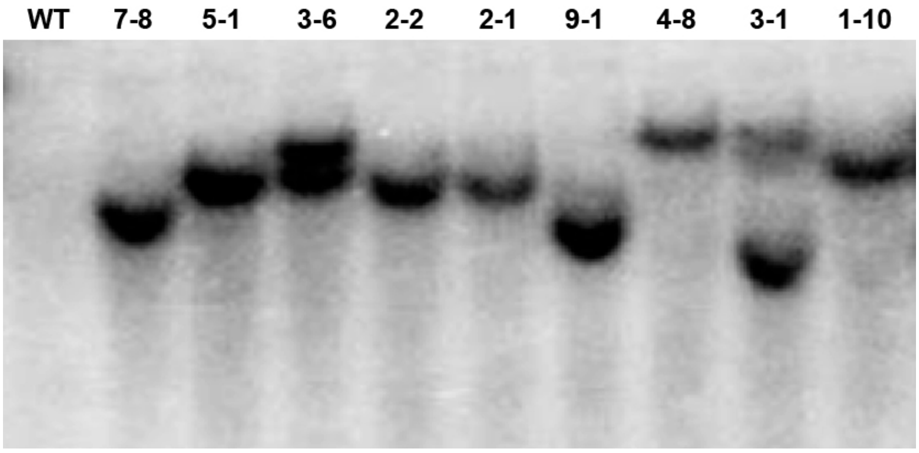

Supplementary Figure 2. Southern blotting of 9 *OsCER1* antisense transgenic lines obtained in this study.
