# Supplementary material for: OsCER1 Plays a Pivotal Role in Very-Long-Chain Alkane Biosynthesis and Affects Plastid Development and Programmed Cell Death of Tapetum in Rice (Oryza sativa L.)
Source: Front Plant Sci. 2018 Sep 6;9:1217. doi: 10.3389/fpls.2018.01217 (PMC6136457; doi:10.3389/fpls.2018.01217)
Supplement: Supplementary file 4 [file Image_3.pdf]

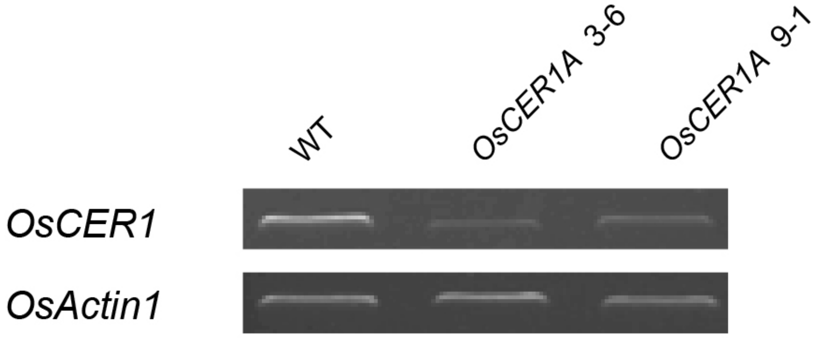

Supplementary Figure 3. Semi-quantitative **RT-PCR analysis of *OsCER1* in the WT and *OsCER1A* lines.**

Expression of *OsCER1* in panicles of the *OsCER1A* 3-6 and *OsCER1A* 9-1 plants. *OsActin1* served as a control.
