# Supplementary material for: OsCER1 Plays a Pivotal Role in Very-Long-Chain Alkane Biosynthesis and Affects Plastid Development and Programmed Cell Death of Tapetum in Rice (Oryza sativa L.)
Source: Front Plant Sci. 2018 Sep 6;9:1217. doi: 10.3389/fpls.2018.01217 (PMC6136457; doi:10.3389/fpls.2018.01217)
Supplement: Supplementary file 5 [file Image_4.pdf]

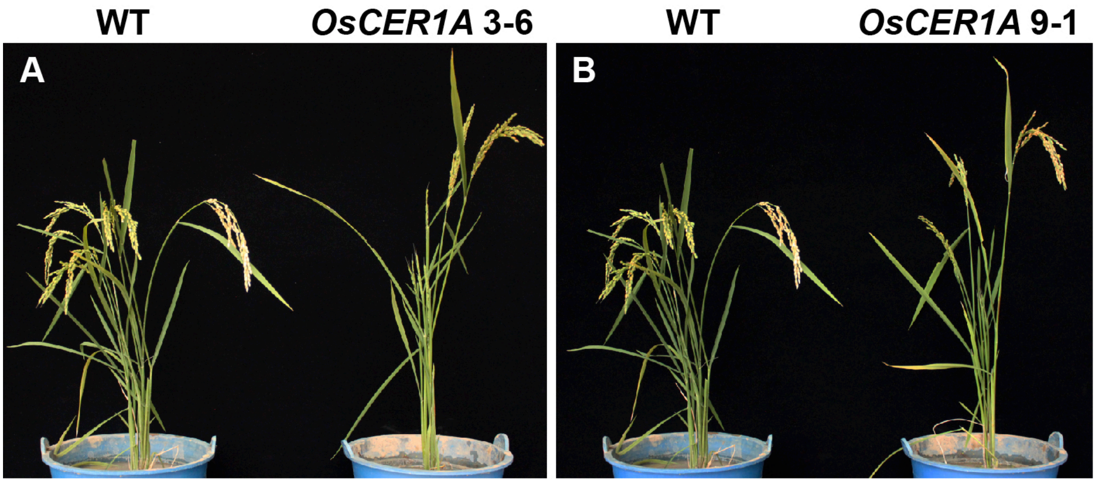


**Supplementary Figure 4. Comparison of the WT plants and *OsCER1A* lines.**
**(A)** Comparison of the WT and *OsCER1A* 3-6 plants. **(B)** Comparison of the WT and *OsCER1A* 9-1 plants.
