# Supplementary material for: OsCER1 Plays a Pivotal Role in Very-Long-Chain Alkane Biosynthesis and Affects Plastid Development and Programmed Cell Death of Tapetum in Rice (Oryza sativa L.)
Source: Front Plant Sci. 2018 Sep 6;9:1217. doi: 10.3389/fpls.2018.01217 (PMC6136457; doi:10.3389/fpls.2018.01217)
Supplement: Supplementary file 6 [file Image_5.pdf]

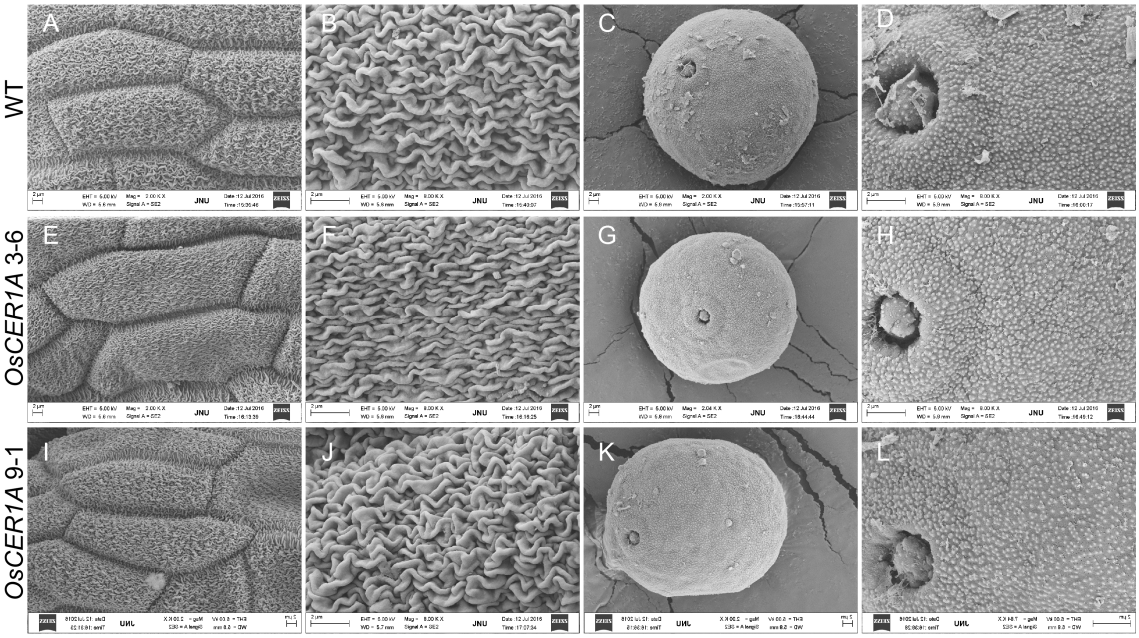

Supplementary Figure 5. Scanning electron microscopy observation of anther walls and mature pollens.

(A), **(E)** and **(I)** Outer surface of mature anther walls of the WT **(A)**, *OsCER1A* 3-6 **(E),** and *OsCER1A* 9-1 **(I)** plants. **(B)**, **(F)** and **(J)** Higher magnifications of **(A)**, **(E)** and **(I)** showing anther wall waxes. **(C)**, **(G)** and **(K)**, Mature pollens of the WT **(C)**, *OsCER1A* 3-6 **(G),** and *OsCER1A* 9-1 **(K)** plants. **(D)**, **(H)** and **(L)** Higher magnifications of **(C)**, **(G)** and **(K)** showing the pollen exine surrounding pollen apertures. Bars = 2 μm.
