# Supplementary material for: OsCER1 Plays a Pivotal Role in Very-Long-Chain Alkane Biosynthesis and Affects Plastid Development and Programmed Cell Death of Tapetum in Rice (Oryza sativa L.)
Source: Front Plant Sci. 2018 Sep 6;9:1217. doi: 10.3389/fpls.2018.01217 (PMC6136457; doi:10.3389/fpls.2018.01217)
Supplement: Supplementary file 7 [file Image_6.pdf]

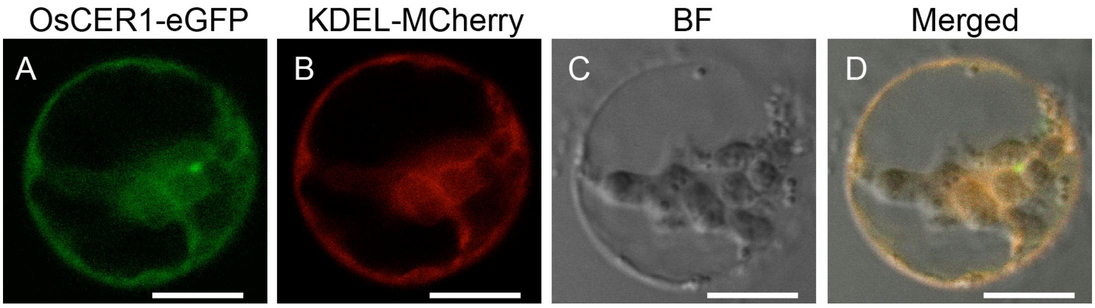

Supplementary Figure 6. Subcellular localization of OsCER1.

(A) A rice protoplast expressing OsCER1(signal peptide)-eGFP showing the green fluorescent signals.

(B) The same rice protoplast cell of **(A)** expressing the KDEL-MCherry showing the red fluorescent signals.

(C) The same rice protoplast cell of **(A)** in bright field (BF).

(D) The merged signal of **(A)**, **(B)**, and **(C)**. Bars = 20 μm.
