# Supplementary material for: OsCER1 Plays a Pivotal Role in Very-Long-Chain Alkane Biosynthesis and Affects Plastid Development and Programmed Cell Death of Tapetum in Rice (Oryza sativa L.)
Source: Front Plant Sci. 2018 Sep 6;9:1217. doi: 10.3389/fpls.2018.01217 (PMC6136457; doi:10.3389/fpls.2018.01217)
Supplement: Supplementary file 8 [file Image_7.pdf]

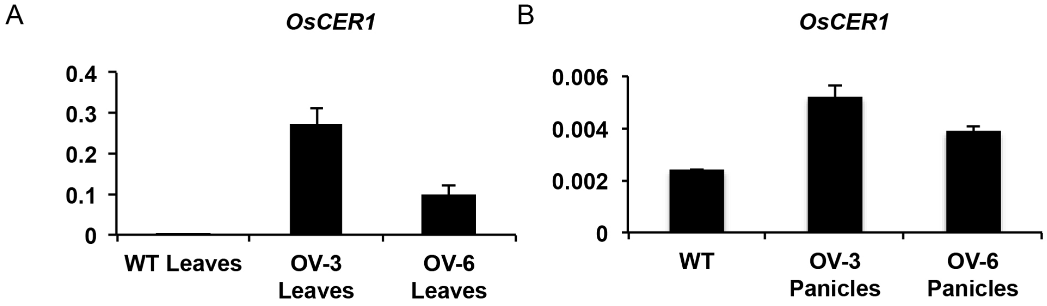

Supplementary Figure 7. Expression analysis in *OsCER1* overexpression lines.

(A) Expression of *OsCER1* examined by qRT-PCR in the leaves of the WT, OV-3 and OV-6 plants.

(B) Expression of *OsCER1* examined by qRT-PCR in the panicles of the WT, OV-3 and OV-6 plants.

OsActin1 served as a control. Each reaction had three biological repeats and error bars indicate the standard deviations (SD).
